# Metabolic drivers of dysglycemia in pregnancy: ethnic-specific GWAS of 146 metabolites and 1-sample Mendelian randomization analyses in a UK multi-ethnic birth cohort

**DOI:** 10.3389/fendo.2023.1157416

**Published:** 2023-05-15

**Authors:** Harriett Fuller, Mark M. Iles, J. Bernadette Moore, Michael A. Zulyniak

**Affiliations:** ^1^ School of Food Science and Nutrition, University of Leeds, Leeds, United Kingdom; ^2^ Public Health Science Division, Fred Hutchinson Cancer Center, Seattle, WA, United States; ^3^ Leeds Institute of Medical Research, University of Leeds, Leeds, United Kingdom; ^4^ Leeds Institute for Data Analytics, University of Leeds, Leeds, United Kingdom

**Keywords:** genetics, GDM, gestational diabetes, South Asian, metabolism, glucose, pregnancy

## Abstract

**Introduction:**

Gestational diabetes mellitus (GDM) is the most common pregnancy complication worldwide and is associated with short- and long-term health implications for both mother and child. Prevalence of GDM varies between ethnicities, with South Asians (SAs) experiencing up to three times the risk compared to white Europeans (WEs). Recent evidence suggests that underlying metabolic difference contribute to this disparity, but an investigation of causality is required.

**Methods:**

To address this, we paired metabolite and genomic data to evaluate the causal effect of 146 distinct metabolic characteristics on gestational dysglycemia in SAs and WEs. First, we performed 292 GWASs to identify ethnic-specific genetic variants associated with each metabolite (P ≤ 1 x 10-5) in the Born and Bradford cohort (3688 SA and 3354 WE women). Following this, a one-sample Mendelian Randomisation (MR) approach was applied for each metabolite against fasting glucose and 2-hr post glucose at 26-28 weeks gestation. Additional GWAS and MR on 22 composite measures of metabolite classes were also conducted.

**Results:**

This study identified 15 novel genome-wide significant (GWS) SNPs associated with tyrosine in the FOXN and SLC13A2 genes and 1 novel GWS SNP (currently in no known gene) associated with acetate in SAs. Using MR approach, 14 metabolites were found to be associated with postprandial glucose in WEs, while in SAs a distinct panel of 11 metabolites were identified. Interestingly, in WEs, cholesterols were the dominant metabolite class driving with dysglycemia, while in SAs saturated fatty acids and total fatty acids were most commonly associated with dysglycemia.

**Discussion:**

In summary, we confirm and demonstrate the presence of ethnic-specific causal relationships between metabolites and dysglycemia in mid-pregnancy in a UK population of SA and WE pregnant women. Future work will aim to investigate their biological mechanisms on dysglycemia and translating this work towards ethnically tailored GDM prevention strategies.

## 1 Introduction

Pregnancy is accompanied by a period of intense maternal metabolic adaptation to meet the energy demands of the foetus (1–3). Mild maternal insulin resistance (IR) is a natural adaption to prioritise adequate glucose for the growing foetus (4). However, if gestational IR exceeds healthy levels and glycaemia is uncontrolled, moderate IR can progress to gestational diabetes mellitus (GDM) (1, 5). GDM is characterised by persistent maternal and foetal exposure to elevated levels of glucose, and places the mother and offspring at risk during pregnancy (i.e., macrosomia and haemorrhaging) and in later life (i.e. from obesity, type 2 diabetes (T2D) and cardiovascular disease) (6).

Globally, GDM is the most common pregnancy complication, affecting up to one in every seven births; however, its prevalence varies between ethnic groups, with South Asian (SA) women at 3-fold greater risk compared to white European (WE) women, irrespective of BMI and country of residence (5, 7). Furthermore, SA women are more likely to develop T2D in later life following a GDM diagnosis (8). Factors driving this disparity in prevalence are not fully understood but metabolism is thought to play a key role (6), given emerging evidence demonstrating (i) differences in metabolic profiles between GDM and non-GDM pregnancies in an ethnic-specific manner (2, 9); and (ii) that a single dietary strategy to manage GDM across all ethnic groups appears ineffective (10, 11). However, heterogeneity of reported metabolite-GDM associations between studies (due to differences in quantification methods, GDM diagnostic criteria (12, 13), ethnic and cultural groups), as well as residual confounding in observational studies have prevented complete understanding and, moreover, advancement to improved equitable care. In short, the field requires a clear and accurate understanding of ethnic-specific metabolic drivers of gestational dysglycemia to inform appropriate and effective prevention and management strategies across ethnic groups.

Mendelian Randomisation (MR) is an instrumental variable technique that can provide estimates of causal associations between an exposure (such as metabolites) and outcome (such as dysglycemia) (14–16). However, no study has yet used MR to establish the presence of casual associations between metabolites and measures of glycaemia at or before the 28 week of pregnancy in an ethnic-specific manner. The present study aimed to address this using the multi-ethnic Born in Bradford (BiB) cohort to identify ethnic-specific metabolic drivers of GDM.

## 2 Material and methods

### 2.1 Exposure data

BiB is a prospective longitudinal birth cohort that aimed to recruit all mothers receiving maternity care in the Bradford Royal Infirmary between 2007-2010 (17). Bradford, a large city in the north of England, has high levels of deprivation and a large SA population, predominantly of Pakistani ancestry. A total of 12,453 women (mean maternal age 27.8) were recruited, 45% of which were of SA ancestry (17, 18). The study was not pre-registered but (SP622) was approved by Born in Bradford. All participants provided written consent and ethnical approval was obtained from the Bradford Research Ethnics Committee (ref07/H1302/112) (17).

Fasted plasma sample collection and high-throughput metabolite quantification by automated NMR (Nightingale Health^©^; Helsinki, Finland) has been previously described and validated to a high accuracy (2). Briefly, samples were taken by trained phlebotomists from BiB participants (26-28 weeks’ gestation) and were processed in the absence of freeze-thaw cycles within 2.5 hours before storage at -80°C. One hundred and forty-six absolute measures of metabolites were included in the analysis following the removal of metabolites expressed as a percentage or ratio to minimise redundancy. In total, 10 overarching classes of metabolites were included in the analysis: lipoproteins (n=97), amino acids (n=9), apolipoproteins (n=2), cholesterols (n=8), fatty acids (n=8), glycerides and phospholipids (n=8), glycolysis related metabolites (n=4), ketone bodies (n=2), measures of fluid balance and inflammation (n=3) and measures of lipoprotein particle diameter (n=3). A full list of included metabolites can be found in **Supplementary Table 1**.

### 2.2 Outcome data

All participants were assesed prior to GDM diagnosis and the 28^th^ week of pregnancy. Individuals were diagnosed with GDM if either their fasting glucose or if 2-hour post-load glucose concentration exceeded 6.1 mmol/L or 7.8 mmol/L following a 75g oral glucose tolerance test (OGTT) (19). The OGTT was performed in the morning following an overnight fast. To maximise power, MR analyses were performed using continuous metabolite values and fasting glucose and 2-hour post glucose. Fasting glucose and 2-hour post glucose values were log normalised prior to analysis.

### 2.3 Metabolite data

Information on metabolite data preparation has been described in full elsewhere (9). In brief, 11,480 blood samples were metabolically profiled from BiB, 54 of which were excluded due to failure of any one of five Nightingale^©^ quality control measures leaving 11,426 samples for imputation. Missing data was imputed *via* multiple imputation using the missMDA package in R (20).

After combining with postprandial glucose data, 3,693 SA and 3,377 individuals whose samples were taken before the 28^th^ week of pregnancy were retained before outlier removal (**Supplementary Figure 1**). Outliers were removed (those outside of 1.5 x IQR) for each metabolite in each ethnicity separately and metabolite values were normalised by taking the log, square root or normal score transformation (NST) as appropriate following the visual inspection of histograms and QQ plots. Following the removal of outliers, the number of individuals available for GWAS analysis of each metabolite varied (**Supplementary Table 2**) but was relatively consistent: for SAs the average sample size for each metabolite was 3622 (range 3472-3688), while for WEs it was 3301 (range 3158-3345). Information on gestational age at sample collection and parity was obtained from obstetric records. Ethnicity was self-reported or obtained from primary care records if missing. Individuals of a SA descent other than Pakistani were excluded from the analysis due to the small sample sizes of these populations. Differences in the distribution of continuous variables between ethnic groups were assessed by Mann-Whitney tests, while differences in the distribution of categorical variables were assessed by chi-squared tests. Women of SA ancestry tended to be older than WE women (27.9 ± 0.1 vs 26.7 ± 0.1 years) and were more likely to be overweight/obese (64.5% vs 53.4%), and be on their ≥2^nd^ pregnancy (67.1% vs 48.8%), but were less likely have smoked during pregnancy (2.9% vs 30.9%) (**Supplementary Table 3**).

### 2.4 Genetic data

Imputed genetic data were obtained from BiB. All samples were genotyped using two chips: the Infinium Global Sequencing Array-24 v.1 (GSA) (~640K SNPs) and the Infinium CoreExome-24 v1.1 BeadChip (~550K SNPs) (21). Genetic data from the Illumina Global Sequencing Array (GSA) and Illumina CoreExome SNPs were combined. Where SNPs were missing in >5% of individuals, they were excluded (21). When evaluating imputed data, the R^2^ value can be a measure of quality control as it reflects to the estimated proportion of genetic variation maintained in the imputed data. As a result, SNPs with an R^2^ <0.9 were excluded prior to analysis.

### 2.5 GWAS analysis

Conventionally a GWAS assumes individuals are unrelated and the inclusion of related individuals can potentially lead to spurious associations (22, 23). However, the removal of individuals from the BiB sample with close ancestry would substantially reduce the sample size. In addition, high rates of consanguinity in the SA stratum of the cohort makes relatedness difficult to assess (24). As such, a GWAS mixed linear model association (MLMA) analysis was conducted in PLINK (version 1.9) that allowed for the inclusion of related individuals (23, 25–27). MLMA models include a fixed effect, adjusted covariates, and an additional random effect comprised of a variance-covariance matrix that models the correlation (here relatedness) between individuals to be accounted for (23, 25). GWAS MLMA models were implemented using the *GCTA* (Genome-wide Complex Trait Analysis) command line tool for each metabolite in both ethnicities (28). To increase power, MLMA-loo (leave-one-out) analysis was used, preventing a SNP from being included in both the fixed and random effects concurrently, thereby avoiding double fitting (25). MLMA models also included parity and principal components (PC) 1 and 2 to account for population stratification (**Supplementary Figure 2**, **Supplementary Table 4**). Gestational age, which showed little variation, was not included in the modelling (median gestational age SA = 184 days, IQR= 182-186.7, median gestational age WE = 184 days, IQR= 182-187). Genomic inflation factors (λ) were calculated for all models for a range of minor allele frequency (MAF) cut-offs (MAF <0.001, 0.001≤ MAF < 0.005, 0.005 ≤ MAF < 0.01, 0.01 ≤MAF < 0.05, 0.05 ≤ MAF < 0.1, and MAF ≥ 0.1) to minimise data loss while also minimising false positives. λ ≥ 1.1 was considered indicative of genomic inflation (29, 30). A MAF cut-off of <0.05 was the least stringent cut-off found to reduce λ to ~1 meaning this cut off was used in the analysis (**Supplementary Table 5**).

When a SNP was found to be associated with a metabolite value in only one ethnicity, a fixed effect inverse-variance weighted meta-analysis was implemented to assess the heterogeneity (via the I^2^ statistic) of identified associations between ethnicities and to see if the SNP retained significance in a larger sample. Meta-analyses were conducted within the command-line tool METAL (31) and supplemented with FUMA (v1.5.2) (32) to investigate SNP function based on their effect on phenotypes.

### 2.6 One-sample MR

#### 2.6.1 Genetic instruments

One-Sample MR was conducted for all 146 metabolite values in both ethnic groups using SNPs identified as significant at a genome-wide suggestive level (p-value ≤ 1 x 10^-5^) in the GWAS. All variants were also entered into MR-base (33) to test for other reported known associations that may be in horizontal or vertical pleiotropy. Metabolites were grouped into their overall classes and SNPs in each class were thinned by linkage disequilibrium (LD) (R^2^<0.2) *via* the NIH LDlink online tool (https://ldlink.nci.nih.gov) reducing the overlap of instruments in each class (34, 35). For individuals of WE ancestry, all European (EUR) and South Asian (SAS) populations in LD link (software that utilises 1000 Genome data) were used to estimate LD due to the expected similarity in their LD structure allowing for an increase sample size and resultant improvement in the accuracy of LD estimates (36). Similarity between 1000 Genome SA samples and BiB samples was assessed using principal components analysis (PCA) in PLINK (version 1.9) because Pakistani samples from BiB originate from a different region of Pakistan (the Mirpur Region) from the 1000 Genome SA samples (26, 27). This is of particular importance in SA as even geographically close populations can have differing allele frequencies due to differing Biraderi (‘Brotherhood’) membership between population subgroups. Biraderi membership is assigned at birth, is an indicator of male lineage as well as social-occupational status which largely governs partner choice and can result in higher levels of consanguinity in the population (21). PCA plots were created using the *ggplot2* package in R studio (version 4.0.2) (37, 38). No clear separation in SA BiB samples and SA 1000 Genome (1000G) samples was identified indicating that LD estimates obtained from 1000G was suitable for use in BiB (**Supplementary Figures 3**-**5**).

#### 2.6.2 Analysis

Genetic Risk Scores (GRS) were created in PLINK (version 1.9) for each metabolite with each SNP receiving a weight based on its estimated effect size on the metabolite obtained from the GWAS (39). One-sample MR was then performed by Two-Stage Least Squares regression (TSLS; *ivpack*, ivreg, and *AER* packages in R version 4.0.2) to obtain a causal estimate for the effect of each metabolite value on the log-normalised continuous measures of fasting glucose and 2-hour post glucose following a 75g oral glucose tolerance test (OGTT) (37, 40, 41). Here, the level of a metabolite is regressed on its respective GRS and, subsequently, the outcome is regressed onto these fitted GRS-metabolite values in the second stage. All MR results have been reported according to STROBE-MR guidelines (42).

When significant associations were identified, leave-one-out analysis was performed. For this, SNPs were removed sequentially from the instrument and changes to the effect estimate and F-statistic was assessed. If the exclusion of a SNP was found to alter either the effect estimates or F-statistic (through the visualisation of forest plots) it is possible that the SNP is influencing the outcome *via* an alternative pathway to other SNPs, potentially highlighting a violation of the 2^nd^ or 3^rd^ MR assumption. To further test for violations of these assumptions, included SNPs were searched for in both the Phenoscanner and GWAS Catalogue databases to identify previously identified associations (43–45) with potential confounders in horizontal (i.e., multiple pregnancies, type-1 diabetes, deprivation index, parity) rather than vertical pleiotropy (i.e., along causal pathway, such anthropometrics). In both databases a p-value ≤ 1 x 10^-5^ was interpreted as indicative of an additional association. Differences between MR and linear regression results were also evaluated *via* the Wu-Hausman statistic to assess deviation of the instrumental variable estimate from the ordinary least squares (OLS) estimate (46). Deviations in these two measures can indicate either confounding in the OLS estimate (indicating a need for MR) or violations of the MR assumptions due to pleiotropy.

#### 2.6.3 *Post-hoc* power analyses

For metabolites that associated with a measure of postprandial glucose in only one ethnic group, *post-hoc* power analyses were performed using the mRnd CNS genomics tool (https://shiny.cnsgenomics.com/mRnd/) to assess whether the absence of an association in the alternate ethnicity was due to limited power (47). Observational and ‘true’ associations required by the tool were obtained by performing linear regression of the outcome on the metabolite and obtaining unadjusted and adjusted estimates (adjusted for maternal age (years), BMI (continuous), smoking status, multiple pregnancy, parity, and gestational age) respectively. Due to the *post-hoc* nature of this analysis, additional power analyses could be conducted assuming the MR estimate to be the true causal effect in the MR calculation. This analysis was performed in the non-significant population for each metabolite associated in only one ethnicity. If power was found to be adequate (80%) at the 5% level (α = 0.05) power was also assessed at the 1% level (α = 0.01).

## 3 Results

### 3.1 GWAS of metabolite measures

A total of 6184 SNPs were associated with at least one metabolite in WEs at the suggestive level (1 x 10^-5^), with 2616 (42.3%) SNPs being associated with a single metabolite measure. However, no SNPs were identified below the genome-wide significant level (p-value <5 x 10^-8^) in WEs.

Fewer SNPs were identified at the suggestive level in SAs, with 3685 SNP-metabolite associations in total, of which 1544 (41.9%) SNPs being associated with only one metabolite measure. SNP associations were identified for 138/146 (94.5%) metabolite exposures in SAs (**Supplementary Table 6**). No SNP was identified as being associated at the suggestive level in both ethnicities, although shared genomic regions were identified between ethnicities (**Supplemental Excel**). Using FUMA to investigate SNP function based on their effect on phenotypes in both ancestries (32), of the 85 genetic variants meta-analysed that surpassed suggestive GWAS significance: 54 were intergenic (i.e., between genes), 21 were intronic (i.e., between exons of a gene), 5 were upstream (i.e., within 250 bps before transcription start site), 3 were downstream (i.e., within 500 bps after transcription start site), and 2 (2%) were exonic (i.e., within protein coding region).

To evaluate the possibility of shared genomic predictors of metabolites, a pooled meta-analysis of effect estimates in both ethnicities was performed. For 90 metabolite values, no associations were found to exceed the genome-wide suggestive level (p<10^-5^) following meta-analyses of both ethnicities. SNP associations were identified at the suggestive level for four metabolite measures (concentration of XL-HDL, total lipids in M-VLDL, mean density of VLDL and citrate) despite these differing in direction of effects in SAs and WEs. In addition, 4 SNPs were associated with alanine, despite these SNPs initially being associated with alanine only in the SA population. These SNPs (rs12256633, rs17121228, rs7096521, rs12240368) are all found on chromosome 10 in the receptor gene *SORCS1* and have not been associated with alanine levels previously (48, 49).

### 3.2 MR Results

After LD thinning, genetic instruments were available for all 146 metabolites in WEs and for 136/146 (93.2%) metabolites in SAs. In WEs, 1040 SNPs were retained following LD thinning including 423 (40.67%) that were unique to an individual metabolite. Fewer SNPs were identified in SAs, where 383 SNPS remained after LD thinning, 195 (50.9%) of which were unique to a single metabolite. Only 2.7% of included genetic instruments (4 metabolites) in WEs and 12.5% (9 metabolites) of included genetic instruments in SAs had an F-statistic < 10, indicating that most instruments were at low risk of weak instrument bias (50). The average F-statistic for WEs instruments was 72.4, while in SAs it was considerably lower at 26.7 (**Supplementary Figure 6**). Screening of genomic predictors using Phenoscanner and GWAS databases did not raise major concerns for horizonal pleiotropy (**Supplementary Table 7**) but did suggest that modification of anthropometrics is a common pathways by which metabolites elicit their effect on dysglycemia − i.e., vertical pleiotropy. However, where horizontal pleiotropy was a possibility (e.g., cholesterol levels), sensitivity analyses were performed (*see 3.2.1.1 and 3.2.2.1 Sensitivity Analyses*). Using MR-Base, we report that [in agreement with recent GWAS (51)], almost all SNPs included in an instrument have been previously associated with dysglycemia metrices or diabetes (**Supplementary Table 8**). However, since most evidence of genomics-diabetes associations are sourced from non-SA populations, these results may not accurately reflect genetic associations in SAs.

#### 3.2.1 White Europeans

Two metabolite values, leucine and mean density of HDL lipoproteins (HDL_D), were associated with both fasting glucose and 2-hour post glucose (**Table 1**; **Supplementary Figures 7, 8**). Specifically, a 1mmol/L increase in blood leucine associated with lower fasting glucose (-0.193 mmol/L, 95% CI -0.069, -0.319) and 2-hour post glucose (-0.443 mmol/L, 95% CI -0.113, -0.774). Likewise, a 1nm increase in mean diameter of HDL associated with lower fasting glucose (-0.082 mmol/L, 95% CI 0.026, 0.138) and 2-hour post glucose (-0.191, 95% CI 0.043, 0.339 mmol/L). No other metabolites were associated with both measures of glucose in WEs.

**Table 1:** Significant MR results in white Europeans.

	Class	Metabolite	Outcome	F statistic	β estimate (95% CI)	WuH
Lower Glucose	S-LDL	S-LDL-P	2-hour post	41.7	-1000 (-20, -1984)	0.017
	Amino Acids	Leucine	Fasting glucose	67.3	-0.193 (-0.069, -0.319)	0.005
			2-hour post		-0.443 (-0.113, -0.774)	0.008
	M-HDL	M-HDL-CE	Fasting glucose	62.4	-0.327 (-0.069, -0.586)	0.043
		M-HDL-C	Fasting glucose	117	-0.189 (-0.021, -0.358)	0.117
Increase Glucose	Lipoprotein Density	HDL_D	Fasting glucose	131	0.082 0.026, 0.138)	0.004
			2-hour post		0.191 (0.043, 0.339)	0.024
	L-HDL	L-HDL-P	2-hour post	108	220 (41.3, 397)	0.02
		L-HDL_L	2-hour post	131	0.264 (0.062, 0,464)	0.014
		L-HDL-C	2-hour post	120	0.279 (0.012, 0.544)	0.048
	Cholesterol	HDL2C	2-hour post	103	0.288 (0.007, 0.583)	0.025
		HDLC	2-hour post	90.6	0.296 (0.007, 0.583)	0.047
		HDL3C	2-hour post	66.6	1.58 (0.002, 3.15)	0.074
	XL-HDL	XL-HDL-CE	2-hour post	109	0.541 (0.079, 1.00)	0.039
	XSVLDL	XS-VLDL-TG	2-hour post	87.8	0.841 (0.098, 1.58)	0.042
	S-HDL	S-HDL-CE	2-hour post	41.9	1.78 (0.448, 3.11)	0.007

Glucose measures are expressed as mmol/L. HDL_D, mean diameter of HDLs (nm); HDLC, total cholesterol in HDL (mmol/L); HDL2C, total cholesterol in HDL2 (mmol/L); HDL3C, total cholesterol in HDL3 (mmol/L); L-HDL-C, total cholesterols in L-HDL (mmol/L); L-HDL_L, total lipids in L-HDL (mol/L); L-HDL-P, concentration of L-HDL (mol/L); M-HDL-C, total cholesterol in M-HDL (mmol/L); M-HDL-CE, cholesterol esters in M-HDL (mmol/L); S-HDL-CE, cholesterol esters in S-HDL (mmol/L); S-LDL-P, concentration of S-LDL (mol/L); XL-HDL-CE, cholesterol esters in XL-HDL (mmol/L); XS-VLDL-TG, triglycerides in XSVLDL (mmol/L); WuH, Wu-Hausman p-value.

For fasting glucose, an increase of 1mmol/L total cholesterol in M-HDL (M-HDL-C) and cholesterol esters in M-HDL (M-HDL-CE) were associated with lower fasting glucose measures (-0.189 mmol/L, 95% CI -0.021, -0.358, and -0.327 mmol/L, 95% CI -0.069, -0.586 respectively). For 2-hr post-glucose, 8 metabolite values were positively associated with this (HDLC, HDL2C, HDL3C, triglycerides in XS-VLDL, cholesterol esters in XL-HDL, total concentration of L-HDL, total lipids in L-HDL and cholesterol esters in S-HDL) and one (total concentration of S-LDL) was negatively associated (**Table 1**). Cholesterol metabolites, measures of total cholesterols in lipoproteins and total cholesterols in lipoproteins were the most common types of metabolite class to be associated with postprandial glucose in WEs, with leucine being the only amino acid identified. Wu-Hausman p-values < 0.05 indicate deviations of the instrumental variable estimate from the OLS estimate (**Table 1**).

##### 3.2.1.1 Sensitivity analyses

For 6 of 13 metabolite values, leave-out one analyses maintained significance (P≤ 0.05) indicating that no individual SNP was driving the identified associations in WEs: leucine, mean diameter of HDL, total lipids in L-HDL, cholesterol esters in S-HDL and cholesterol esters in M-HDL (**Supplementary Figure 9**). For the remaining 8 metabolites, β values were consistent across leave-one-out analyses although not all associations remained significant. Additionally, for 12/13 metabolites (all but M-HDL-CE), the F-statistic did not substantially differ through the exclusion of individual SNPs from the instruments, which suggests they were not substantially driven by a single SNP (**Supplementary Figure 9**). The exception to this was cholesterol esters in M-HDL, where the exclusion of rs2138011 or rs739018 increased the F-statistic.

Three of the metabolites (leucine, L-HDL-L, L-HDL-C) that were associated with postprandial glucose in WEs included a SNP that previous studies have associated (p ≤ 1 x 10^-5^) with at least one potential confounder (BMI, hypertension or waist circumference) (**Supplementary Table 7**). The removal of these SNPs from the instrument did not impact the significance of the associations identified for leucine or L-HDL-L (**Table 2**). However, for L-HDL-C instrument, the exclusion of two SNPs (rs5576825 and rs6811162) previously associated with a potential confounder (waist circumference and hypertension respectively) resulted in non-significant association between L-HDL-C and 2-hour post glucose. Importantly, for both SNPs, it is conceivable that the confounders could reside on their causal pathway (i.e., vertically pleiotropic, where L-HDL-C effects 2-hr post-prandial glucose through its effect on weight gain) rather than be in horizontal pleiotropy and may, therefore, violate the 2^nd^ MR assumption (50).

**Table 2:** Removal of potentially pleiotropic SNPs.

Ethnicity	Metabolite	SNP	Gene	Associated confounder	Initial		Confounder removal	
					β estimate (95% CI)	WuH	β estimate (95% CI)	WuH
**WE**	Leucine	rs2984433	ACTG1P9	BMI, Obesity class 1, weight	-0.193 (-0.319, -0.068) ^FG^	0.004	-0.203 (-0.339, -0.068) ^FG^	0.006
					-0.443 (-0.774, -0.113) ^2H^	0.024	-0.547 (-0.909, -0.185) ^2H^	0.002
	L-HDL_L	rs6811162	ENPEP	Self-reported hypertension, diagnosed high blood pressure	0.264 (0.062, 0.464)	0.014	0.301 (0.092, 0.510)	0.006
	L-HDL-C	rs5576825	LINC01621 ELOVL4	Waist circumference	0.279 (0.012, 0.544)	0.048	0.323 (0.0456, 0.602)	0.107
		rs6811162	ENPEP	Self-reported hypertension, diagnosed high blood pressure			0.241 (-0.037, 0.519)	0.026
		rs5576825 + rs6811162	-	-			0.287 (-0.004, 0.578)	0.063
	XL-HDL class	rs5576825	LINC01621 ELOVL4	Waist circumference	-0.285 (-0.552, -0.018)	0.015	-0.244 (-0.528, 0.040)	0.135
**SA**	LA	rs12720820	APOB	Self-reported high cholesterol, coronary artery disease, treatment with cholesterol lowering medication	0.477 (0.013, 0.939)	0.030	0.335 (-0.982, 0.763)	0.459
	FAw6	rs12720820	APOB	Self-reported high cholesterol, coronary artery disease, treatment with cholesterol lowering medication	0.445 (0.094, 0.794)	0.007	0.223 (-0.398, 0.843)	0.105
	L-HDL-PL	rs7486176	C12orf76	Systolic blood pressure, diagnosed high blood pressure, hypertension	0.692 (0.106, 1.28)	0.021	0.853 (0.170, 1.54)	0.013
	Fatty Acid class	rs12720820	APOB	Self-reported high cholesterol, coronary artery disease, treatment with cholesterol lowering medication	0.172 (0.018, 0.327)	0.018	0.142 (-0.049, 0.334)	0.119

2H, 2-hour post glucose; FAw6, Total n-6 fatty acids; FG, fasting glucose; LA, 18,2 linoleic acid (mmol/L); L-HDL-C, total cholesterols in L-HDL (mmol/L); L-HDL_L, total lipids in L-HDL (mmol/L); L-HDL-PL, phospholipids in L-HDL (mmol/L); SA, South Asian; WE, White European; WUH, Wu-Hausman p-value.

#### 3.2.2 South Asians

No metabolite was associated with both fasting glucose and 2-hour post glucose in SAs (**Supplementary Figures 7, 8**). Although, for fasting glucose, a 1 mmol/L increase in either total FAw3 or S-HDL-C was associated with an increase of fasting glucose by 0.432 mmol/L (95% CI 0.063 – 0.798) and 1 mmol/L (95% CI 0.116 – 1.882) respectively. No metabolite associated with a decrease in fasting glucose in SAs.

Nine metabolites associated with 2-hour post glucose levels in SAs. Of these, 4 metabolites, LA, FAw6, total lipids in M-VLDL (M-VLDL-L) and total phospholipids in L-HDL (L-HDL-PL), associated with an increase in with 2-hour post glucose, with the largest effect being identified for L-HDL-PL. Specifically, a 1mmol/L increase in L-HDL-PL associated with a 0.692 mmol/L increase (95% CI 0.106 - 1.280) in 2-hour post glucose. A further 5 additional metabolites were associated with a decrease in 2-hour post glucose: concentration of L-LDL (L-LDL-P), total cholesterols in IDL (IDL-C), cholesterol esters in IDL (IDL-CE) concentration, total cholesterols in IDL (IDL-C), total lipids in small S-LDL (S-LDL-L), and total lipids in small S-HDL (S-HDL-L). The largest decrease in 2-hour post glucose was observed for L-LDL-P where a 1mmol/L increase in L-LDL-P associated with a 3.86 mmol/L decrease (95% 0.467 - 7.27) in 2-hour post glucose levels (**Table 3**).

**Table 3:** Significant MR results in South Asians.

	Class	Metabolite	Outcome	F statistic	β estimate (95% CI)	WuH
Lowers Glucose	L-LDL	LDL-P	2-hour post	11.7	-2238 (-4828 -193.7)	0.024
	S-LDL	SLDL-L	2-hour post	11.1	-3.86 (0.467, -7.27)	0.015
	IDL	IDL-C	2-hour post	10.9	-1.19 (-0.12, -2.27)	0.021
		IDL-CE	2-hour post	11.8	-1.34 (-0.144, -2.55)	0.023
	S-HDL	S-HDL-L	2-hour post	11.4	-1.23 (-0.137, -2.32)	0.012
Increases Glucose	Fatty Acids	LA	2-hour post	20.9	0.477 (0.013, 0.939)	0.030
		FAw3	Fasting glucose	10	0.432 (0.063, 0.798)	0.008
		FAw6	2-hour post	33.4	0.445 (0.094, 0.794)	0.007
	M-VLDL	M-VLDL-L	2-hour post	68.4	0.046 (0.009, 0.083)	0.008
	L-HDL	L-HDL-PL	2-hour post	43.3	0.692 (0.106, 1.28)	0.021
	S-HDL	S-HDL-C	Fasting glucose	22	1 (0.116,1.882)	0.012

Glucose measures are expressed as mmol/L. FAw3, total n-3 fatty acids; FAw6, total n-6 fatty acids; IDL-C, total cholesterols in LDL (mmol/L); IDL-CE, cholesterol esters in LDL (mmol/L); LA, 18,2 Linoleic Acid (mmol/L); LDL_P, concentration of LDL particles (mol/L); L-HDL-PL, phospholipids in L-HDL (mmol/L); M-VLDL-L, total lipids in M-VLDL (mmol/L); S-HDL-C, total cholesterols in S-HDL (mmol/L); S-HDL-L, total lipids in S-HDL (mmol/L); S-LDL-L, total lipids in S-LDL (mmol/L); WuH, Wu-Hausman p-value.

Fatty acids were the class of metabolites most frequently associated with postprandial glucose in SAs. All three fatty acids (LA, FAw3 and FAw6) associations identified in SAs were of similar magnitude: a 1 mmol/l increase of FAw3 associated with a +0.4 mmol/l increase in fasting glucose or and a 1 mmol/increase of FAw6 and LA associated with a +0.4 mmol/l increase of 2-hour post glucose.

No metabolite found to be associated with postprandial glucose measures in WEs was found to be associated with postprandial glucose in SAs. However, in both populations, members of the S-HDL and L-HDL class were found to be associated with increased postprandial glucose.

##### 3.2.2.1 Sensitivity analyses

Six instruments in SAs were comprised of a single SNP meaning it was not possible to perform a leave-one-out analysis for these metabolites. For the remaining 5 metabolites, associations were consistent across each leave-one-out analyses (**Supplementary Figure 9**). Likewise, no large differences in F-statistics following the removal of individual SNPs were identified (**Supplementary Figure 10**).

Just as in WEs, 3 metabolites identified in SAs included SNPs associated with cholesterol or hypertension, which are potential confounders of the association between metabolites and dysglycemia (**Supplementary Table 7**). Significance was maintained following the removal of SNP rs7486176 (found within the *C12orf76* gene) from the total phospholipids in L-HDL instrument. For the LA and FAw6 exposures, the removal of SNP rs12720820 (found within the *APOB* gene) resulted in a non-significant association indicating that this SNP was the main driver of the identified association (**Table 2**). In leave-one-out analyses, the removal of SNP rs58865405 from the FAw6 instrument resulted in non-significance, although the biological role of this SNP remains unknown.

### 3.3 *Post-hoc* analysis: analysis of metabolite classes

Numerous SNPs were found to be associated with more than one metabolite measure, particularly for metabolites in the same metabolite class (**Supplementary Figures 11**-**12**). This was anticipated since many metabolomic pathways are biologically intertwined. To minimise the risk of violation of the third MR assumption (that the genetic instrument must only influence the outcome *via* the exposure and not *via* an alternative biological pathway) (14), the collective effect of an entire class of metabolites on postprandial glucose measures was examined. A composite score for each metabolite class was created by placing all metabolites in a single class (e.g. all LDL metabolites), conducting a PCA and extracting PC1. This was only possible for 20 classes in WEs and 21 classes in SAs that had > 2 metabolites and ≥70% of the class variation was explained by PC1 (**Supplementary Table 9**). To assess the impact of outliers on PCA, outliers were defined and removed based on two cut-offs: standard (1.5 X IQR from the median) and stringent (3 x IQR from the median). For all classes, PC1 and PC2 scores were comparable after removal of both types of outliers so only 3xIQR outliers were removed prior to analyses (**Supplementary Table 10**).

138 SNPS remained after LD thinning in WEs, 87 (63.04%) of which were unique to a single metabolite class exposure. 19/20 (95%) of the metabolite classes examined in WEs had an F-statistic ≥10 (the only exception being the MHDL class). 54 SNPS remained after LD thinning in SAs, 42 (77.78%) of which were unique to a single metabolite class. Screening of metabolite class predictors in Phenoscanner and GWAS databases did not raise concerns for horizonal (**Supplementary Table 11**). Despite the lower number of SNPs identified in SAs,17/20 (85%) of the metabolite classes examined had a F-statistic ≥10 indicating that instrument strength was still sufficient, for all classes except for the non-branched amino acids, LLDL class, and all VLDL classes. On average, the mean F-statistic of metabolite class instruments was 19.83%. On average genetic instruments of composite measures of the metabolite classes were weaker than the instruments for individual metabolites in both WEs and SAs (**Supplementary Figure 13**).

In WEs, 4 metabolite classes were associated with a glucose measure: S-LDLs associated with fasting and 2-hour post glucose; M-LDL and all LDLS (i.e. the collective grouping of HDLs, MDLs and SDLs) were associated with fasting glucose, and XL-HDLs were associated with 2-hour post glucose (**Table 4**). In SAs, the fatty acid metabolite class were associated with 2-hour post glucose levels. No other associations were identified in SAs.

**Table 4:** Significant MR results from the analysis of metabolite classes.

Ethnicity	Metabolite class	Outcome	F statistic	β estimate (95% CI)	WUH
**White European**	XL-HDL	2-hour post glucose	96.2	-0.285 (-0.018, -0.552)	0.015
	M-LDL	Fasting glucose	95.5	-0.048 (-0.004, - 0.091)	0.024
	S-LDL	Fasting glucose	98	0.084 (0.007, 0.162)	0.024
		2-hour post glucose	98	-0.249 (-0.016, -0.482)	0.066
	All LDL	Fasting glucose	95	0.038 (-0.005, -0.068)	0.016
**South Asian**	Fatty Acids	2-hour post glucose	32.8	0.172 (0.018, 0.327)	0.0184

Glucose measures are expressed as mmol/L. WuH: Wu-Hausman p-value.

As with the sensitivity analyses of individual metabolites, the removal of individual SNPs was not found to greatly impact the F-statistic of most instruments (**Supplementary Figures 14**-**15**). However, for the fatty acid metabolites class, the exclusion of rs12720820 or rs7159441, and for ‘XL-HDL’, the removal of rs55768285, resulted in non-significant associations, suggesting that these SNPs are key drivers of the association. (**Table 4**; **Supplementary Table 10**).

### 3.4 Power analysis

R^2^ values were consistently lower in the ethnic group where an effect was not detected but all genetic instruments for the metabolite values had an F-statistic ≥ 10, indicating that weak instrument bias was not responsible for the absence of significant effects. Where an association was identified in one ethnic group but not another, to determine whether the absence of an association was potentially due a lack of power in the other ethnicity rather than an ethnic-specific effect, *post-hoc* power analyses were performed.

When using MR estimates as an estimate for the true causal effect both the analyses of FAw3 and the overall fatty acid class in WEs were adequately powered to detect the observed MR effect in both populations. Therefore, the absence of an effect of FAw3 in WEs is unlikely due to inadequate power (**Supplementary Table 12**). The analysis of HDL2C and HDL3C in SAs was also sufficiently powered to detect the observed MR effect in WEs.

## 4 Discussion

This study has identified ethnically distinct associations between a range of metabolites and postprandial glucose measures taken during pregnancy in SAs and WEs, with notably no shared associations were identified. Fourteen metabolites were found to be associated with postprandial glucose measures in WEs. Whereas, a distinct set of 11 metabolites were associated in SAs. In WEs, cholesterols and lipoproteins were the metabolite classes associated with postprandial glucose measures, while in SAs fatty acids were the most commonly associated.

Furthermore, through an extensive GWAS of metabolites, this study identified novel genome-wide significant associations in relation to acetate (1 SNP, rs10945476) and tyrosine (15 SNPs, all on chromosome 17) in SAs. No previous associations have been identified for SNP rs10945476, found within the non-coding transcript gene *PRDM15* in relation to acetate or any other exposure.

Interestingly, 3 of the 15 SNPs associated with tyrosine are found in a transmembrane transporter gene, *SLC13A2*. Moreover, an additional 10 of the newly identified 15 SNPs associated with tyrosine were found in the *FOXN* gene, a transcription factor that has previously been identified to be associated with ceramide levels (a lipid metabolite) in a GWAS from a Chinese cohort (52). Moreover, ceramide has been shown to induce tyrosine phosphorylation in membrane proteins meaning it is plausible that a gene associated with ceramide is also associated with tyrosine levels in an Asian population (51). Interestingly, ceramide has been proposed as a mediator of the interaction between saturated fat and insulin resistance and has been associated with T2D and cardiovascular disease (53). To the best of our knowledge tyrosine levels have not previously been associated with either *FOXN SLC13A2*. The remaining 2 of the 15 SNPs identified as being associated with tyrosine in SAs, are currently not in any known genes. All 15 SNPs identified as being associated with tyrosine in SAs are in LD with each other in 1000G SA populations (all R^2^ ≥ 0.38). In agreement with a recent GWAS of GDM and T2D (54), almost all SNPs included in an instrument significantly associated with either outcome (fasting glucose or 2-hour post glucose) have previously been associated with a diabetes related disease outcome, providing additional evidence for their validity as instrumental variables. However, since most evidence of genomics-diabetes associations are sourced from non-SA populations, these results may not accurately reflect genetic associations in SAs.

### 4.1 Identified associations in white Europeans (WEs)

#### 4.1.1 Leucine

Branched chain amino acids (BCAAs), including leucine, are predominantly metabolised in skeletal muscles where they regulate protein synthesis and mitochondrial functions (55). In addition, BCAAs are hormonal signalling regulators and are expected to module insulin resistance (IR) through increasing insulin secretion in human pancreatic β-cells (56, 57). Our study found leucine to be negatively associated with both fasting glucose and 2-hour post glucose levels during pregnancy in WEs; with 1 mmol/L of leucine associated with a decrease of 0.193 mmol/L in fasting glucose and 0.327 mmol/L 2-hour post glucose respectively. Although few studies have investigated the role of leucine in glucose regulation during pregnancy, interestingly the ratio of leucine/isoleucine was similarly found associated with reductions in fasting glucose in the HAPO study, a multi-ethnic cohort of pregnant women of Afro-Caribbean, Mexican American, Northern European, and Thai ancestry (58). Common dietary sources of leucine include meat products and cheese, with smaller amounts also being present in other dairy products (such as dairy and yoghurt), fish and in certain legumes and nuts, such as dried raw broad beans and pine nuts (55). Hence, dietary interventions aimed at increasing leucine levels during pregnancy, possibly through a dietary intervention promoting the consumption of lean animal protein, low-fat dairy and nuts, may help improve pregnancy hyperglycaemia in WEs.

#### 4.1.2 Cholesterols

HDL cholesterol is colloquially described as ‘good cholesterol’ due to its role in the removal of cholesterols from atherosclerotic plaque, thereby reducing an individual’s risk of CVD (59). Furthermore, low HDL levels have commonly been associated with diabetes in humans, with HDL shown to increase insulin secretion and β-cell survival (60, 61). We identified four associations between HDL cholesterol and postprandial glucose measures in WEs. Herein, 1 mmol/L increase in S-HDL-CE confers a 1.78 mmol/L increase (95% CI 0.49 – 3.11) in 2-hour post glucose. This is consistent with previous evidence from a Finnish sample of overweight and obese women where cholesterol esters in S-HDL were higher in the serum samples of GDM cases at ~14 weeks gestation (62). Discrepancies in the direct effect of HDL cholesterol on dysglycemia have also been identified in the genetic literature (61), with a recent review highlighting that while a genetic study utilising linear relation analysis did find HDLs to have a protective effects against T2D (n cases = 2,447) (63), the same effect was not been replicated in an MR setting (n cases= 47,627) (64). When considering LDL cholesterols, only S-LDLs was found to be significantly associated with a postprandial glucose measure (fasting glucose) in WEs. Additionally, in our composite analysis of metabolite classes, S-LDLs were associated with fasting glucose and 2-hour post glucose in WEs, whereas the M-LDL and all LDL (a combined measure of S-LDLs, M-LDLs and HDLs) classes were associated with fasting glucose. Unfortunately, because composite scores were comprised of PC1 coordinates the direction of effect of these associations could not be evaluated. To our knowledge, no previous study has conducted an MR of metabolites on dysglycemic predictors of GDM.

#### 4.1.3 Triglycerides

Triglycerides are an abundant class of lipid particles found in the blood, originating from either from the consumption of dietary fats or as a result of hepatic metabolism (65, 66). Once in the blood, triglycerides can be incorporated into HDL and LDL cholesterol particles. In addition to dietary triglyceride consumption, dietary fatty acids can be converted into triglycerides before they enter circulation, highlighting the complex relationship between triglyceride, cholesterol, and fatty acid levels (65).

Our results suggest triglycerides in XSVLDL (XS-VLDL-TG) associate with increased 2-hour post glucose (0.841 mmol/L) in WEs. In agreement with these findings, increased triglycerides in XSVLDL levels have also previously been associated with increased likelihood of GDM in a Finnish population (62). No other triglyceride was found to be associated with in WEs. One explanation no additional associations were detected could be due to the average BMI of the WEs in BiB. For example, an analysis of a prospective Irish cohort (~94% WE) found that triglyceride levels were only associated with GDM in obese individuals, a higher average BMI than that observed in the BiB cohort (67). Further confirmation of these findings of increased triglycerides in XS-VLDLs would suggest that this association is, at least in part, responsible for the identified associations between diets high in fats and increased prevalence of GDM in WEs (10).

### 4.2 Identified associations in South Asians (SAs)

#### 4.2.1 Fatty acids

Polyunsaturated acids (PUFAs) are consumed in the diet and can be converted to long-chain PUFAs (LC-PUFAs) through a process of desaturation and elongation reactions that predominately occur in the liver (68). Changes in dietary patterns can have a large impact on fatty acid composition in the body and with-it disease risk. For example, a western dietary pattern, which has high levels of n-6 fatty acids, has been associated with GDM risk (10, 69). In a cohort of Chinese adults, total n-6 fatty acids and 18:2 n-6 levels at baseline in venous blood samples were both found to associate with an increased risk of T2D after ~8 years of follow up, while increased n-3 fatty acid levels were protective (70). However, a recent two-sample MR suggested only a negligible effect of n-6 PUFA synthesis on T2D in a predominantly WE cohort (71). Moreover, the relationship between n-3, n-6, n-9 fatty acids and GDM remains inconclusive, and in a recent (2021) systematic review none of the identified studies (n=15) was conducted in a SA population (69), highlighting the need for more studies exploring the role of fatty acids in GDM development in Asians (72).

This study provides evidence of an association between LA and total FAw6 levels and an increase in 2-hour post glucose levels during pregnancy in SAs. In addition, the fatty acid class associated with 2-hour post glucose in SAs. Through a leave-out-one sensitivity analyses for the FAw6 and LA instruments, the removal of the SNP rs12720820 (found within the *APOB* gene) resulted in non-significant associations for both exposures, indicating that this rs12720820 was the largest contributor to the identified associations and has previously been associated with cholesterol levels and the use of cholesterol lowering drugs (73). Interestingly, FAw3 also associated with increased 2-hour post glucose in SAs; however, with only one SNP potential pleiotropy could not be explored.

**Figure 1:**
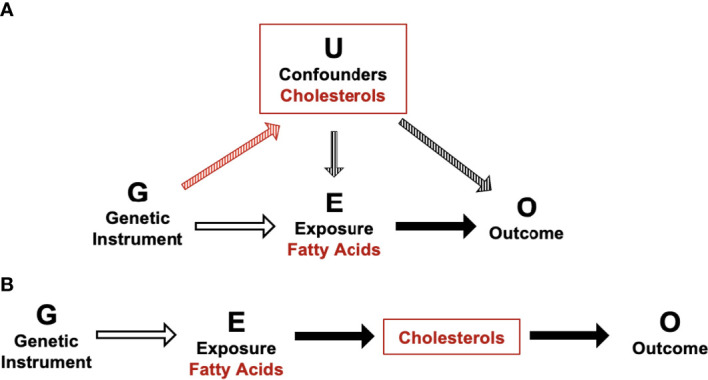
Schematic of potential horizontal and vertical pleiotropy in relation to fatty acid and cholesterol metabolites and postprandial glucose measures. **(A)** Illustration of horizontal pleiotropy. **(B)** Illustration of vertical pleiotropy. Vertical pleiotropy does not result in a violation of the 2nd MR assumption because the metabolites progress along a single linear causal pathway.

It is well established that fatty acid profiles can impact blood cholesterol levels (74–76). In addition, increased dietary cholesterol has previously been associated with an increased risk of GDM in a systematic review of observational studies (77). Taken together, our data confirm that fatty acids and cholesterol metabolites are in vertically pleiotropy and are likely impacting gestational dysglycemia *via* the same causal pathway. Unlike horizontal pleiotropy, vertical pleiotropy does not result in a violation of the 2^nd^ MR assumption as cholesterol is not acting as a confounder, meaning MR estimates are still valid (**Figure 1**). Furthermore, it is also possible that this interaction between fatty acids and cholesterols may be ethnic-specific due to the absence of associations identified between fatty acids and postprandial glucose measures in WEs. In addition to possible variations in cholesterol metabolism, it is plausible that variations in fatty acid synthesis are also partially responsible for the increased GDM risk experienced by SAs. For example, variants within the *FADS* genes impact LC-PUFA conversion (78, 79). Current evidence suggests that SAs are likely to synthesise LC-PUFA more quickly than WEs, which could contribute to elevated risk of prolonged exposure to elevated LC-PUFA levels (namely, w6) and risk of dysglycemia (78, 79). If these ethnic differences in fatty acid metabolism are confirmed to be linked to disease risk, it would aid in the development of tailored GDM prevention strategies that focus on modifying fatty acid profiles in an ethnic-specific manner.

The analyses found no association between triglycerides and dysglycemia in SAs. This agrees with a recent meta-analysis that concluded, although triglyceride levels associated with likelihood of GDM (I^2^ ≥ 84%) (80), after stratification by culture/geographical location, they found no association between triglyceride levels and likelihood of GDM. The reasons for this are unclear but it has also been shown that SAs have a higher prevalence of hypertriglyceridemia than WEs and at lower BMI levels, meaning it is possible that the difference in triglyceride levels and in SA GDM cases and controls is less pronounced than in WEs (81).

### 4.3 Strengths and limitations

This analysis has several strengths. Firstly, this study involved a large and comprehensive panel of metabolites allowing for the relationships between metabolites and postprandial glucose to be thoroughly investigated. Secondly, this is the first MR study to investigate dysglycemia during pregnancy while also being one of the few MR studies to be conducted in a SA population. Finally, through leave-one-out analyses and the searching of both Phenoscanner and GWAS Catalog databases, violations of the 2^nd^ and 3^rd^ MR assumptions were thoroughly investigated meaning that it was possible to conclude that identified robust associations between genetic variants and outcomes (1^st^ assumption of MR) may not be subjected to horizontal pleiotropy and that identified causal associations are valid due to the absence of detectable violations of the MR assumptions.

Nonetheless, this study has some limitations. Firstly, metabolites are highly correlated meaning it is not possible to confidently interpret that an individual metabolite is independently associated with a postprandial outcome measure. To account for this limitation MR analyses were performed on composite measures of each metabolite class (when PC1 explained ≥70% of the variation in the metabolite class) to assess the overall impact of each metabolite class on pregnancy dysglycemia. Secondly, MR also assumes the level of genetically conferred exposure from conception to the time of measurement is constant, which is unknown when studying metabolites − therefore, we cannot presume that these associations would be observed outside of pregnancy. Thirdly, limited sample size may have led to some underpowered analyses and combined with high consanguinity persuaded us to use statistical modelling to account for ‘relatedness’ (rather than participant pruning) to preserve analytical power and study integrity. We acknowledge that this strategy has limitations, and tested for their effect (i.e., LOO analysis) (25), and look forward to the future when larger SA prospective cohort are available, and we can validate these results with increased confidence. In addition, the limited sample size meant that further adjustment could not be made at the GWAS stage since missing data in certain variables would further reduce sample size (e.g., age) and some associations may have been underpowered to detect an effect. However, a *post hoc* power analyses found that for some metabolite values significant effects only identified in one ethnicity were possible to detect in the alternate ethnicity. Fourthly, some genetic instruments included only one SNP meaning it was not possible to evaluate the impact of pleiotropy for any identified associations involving these instruments. Fifthly, it was not possible to fully assess the presence of associations between SNPs included in significant instruments, potential confounders and T2D traits in SAs due to the limited number of GWAS conducted in SAs. Although it is likely that many of the associations in WEs are also present in SAs, it is possible that not all associations identified in WE are present in SAs and that some SA-specific pleiotropic associations are unknown. Lastly, due to limitations in data availability in SAs a two-sample MR could not be conducted meaning it was not possible to assess the generalisability of these findings.

## 5 Conclusions

The presence of causal relationships between a comprehensive set of distinct metabolites and metabolite families with postprandial glucose measures (fasting glucose and 2-hour post glucose) in mid-pregnancy has been established in a UK SA and WE population. This study has found a range of metabolite values to be associated with postprandial glucose measures in WEs and high-risk SA women, although more associations were identified in WEs despite these individuals being at lower risk of GDM. In high-risk SA women, total n-6 fatty acids and the n-6 fatty acid, LA appear to increase postprandial glucose levels suggesting that fatty acids may be responsible for a large proportion of metabolically driven risk for GDM experienced by this population. Future work in a larger sample (potentially using a two-sample MR) and a larger panel of metabolites is needed to investigate our findings and hypotheses more closely, ideally over the course of a pregnancy in order to aid in GDM prevention in this high-risk population.

## Data availability statement

Publicly available datasets were analyzed in this study. This data can be found here: Born in Bradford [https://borninbradford.nhs.uk].

## Author contributions

HF, MI, and MZ contributed to conception and design of the study. HF organized the database and performed the statistical analysis. JM, MI, and MZ supervised the study. HF and MZ wrote the first draft of the manuscript. All authors contributed to the article and approved the submitted version.
