# Supplementary material for: Metabolic drivers of dysglycemia in pregnancy: ethnic-specific GWAS of 146 metabolites and 1-sample Mendelian randomization analyses in a UK multi-ethnic birth cohort
Source: Front Endocrinol (Lausanne). 2023 May 15;14:1157416. doi: 10.3389/fendo.2023.1157416 (PMC10225646; doi:10.3389/fendo.2023.1157416)
Supplement: Supplementary file 2 [file DataSheet_2.docx]

**Supplementary: Metabolic drivers of dysglycemia in pregnancy: A GWAS of metabolites and one-sample Mendelian Randomisation study in a UK multi-ethnic birth cohort.**

Authors: Harriett Fuller, Mark M Iles, J. Bernadette Moore, Michael A. Zulyniak.

*Corresponding Author: Michael Zulyniak. Email. m.a.zulyniak@leeds.ac.uk.

Table of Contents

[Supplementary Figure 1: Born in Bradford sample selection. 3](#_Toc130547537)

[Supplementary Figure 2: PCA to account for population stratification. 4](#_Toc130547538)

[Supplementary Figure 3: PCA comparing BiB data to all SA data from 1000G. 5](#_Toc130547539)

[Supplementary Figure 4: PCA comparing BiB data to all data from 1000G. 6](#_Toc130547540)

[Supplementary Figure 5: PCA comparing BiB data to all data from 1000G with the Pakistani BiB and South Asian 1000G populations. 7](#_Toc130547541)

[Supplementary Figure 6: Number of SNPs and the strength of in each instrument. 8](#_Toc130547542)

[Supplementary Figure 7a: Forest plots of β values following leave-one-out analyses for white Europeans. 9](#_Toc130547543)

[Supplementary Figure 7b: Forest plots of β values following leave-one-out analyses for white Europeans. 10](#_Toc130547544)

[Supplementary Figure 8: F statistics following leave-one-out analyses for identified associations in white Europeans. 11](#_Toc130547545)

[11](#_Toc130547546)

[Supplementary Figure 8 continued: F statistics following leave-one-out analyses for identified associations in white Europeans. 12](#_Toc130547547)

[Supplementary Figure 9: Forest plots of β values following leave-one-out analyses for identified associations in South Asians. 13](#_Toc130547548)

[13](#_Toc130547549)

[Supplementary Figure 10: F statistics following leave-one- out analyses for identified associations in South Asians. 14](#_Toc130547550)

[Supplementary Figure 11: Overlap of SNPs identified by class in white Europeans. 15](#_Toc130547551)

[Supplementary Figure 12: Overlap of SNPs identified by class in South Asians. 16](#_Toc130547552)

[Supplementary Figure 13: Number of SNPs and strength of each instrument in the analysis of each metabolite class. 17](#_Toc130547553)

[Supplementary Figure 14: Forest plots of β values following leave-one-out analyses for identified associations in the analysis of metabolite class. 18](#_Toc130547554)

[Supplementary Figure 15: F statistics following leave-one-out analyses for identified associations in the analysis of metabolite classes. 19](#_Toc130547555)

[19](#_Toc130547556)

[Supplementary Table 1: List of metabolite values included within this study. 20](#_Toc130547557)

[Supplementary Table 2: Proportion of outliers identified in each ethnicity. 24](#_Toc130547558)

[Supplementary Table 3: Characteristics of included Born in Bradford (BiB) participants by ethnicity 27](#_Toc130547559)

[Supplementary Table 4: Regions of high linkage disequilibrium (LD) excluded from PCA of genetic data 28](#_Toc130547560)

[28](#_Toc130547561)

[Supplementary Table 5: Absolute deviations in λ from 1 in each ethnicity. 29](#_Toc130547562)

[Supplementary Table 6: Number of SNPs identified in each ethnicity 30](#_Toc130547563)

[Supplementary Table 7: Investigation of pleiotropy in the GWAS Catalogue and Phenoscanner databases for individual metabolite measures. 33](#_Toc130547564)

[Supplementary Table 8: Associations of SNPs in Significant Instruments with Diabetes Traits in MR BASE. 39](#_Toc130547565)

[Supplementary Table 9: Percentage of variation explained by PC1 in each metabolite class. 45](#_Toc130547566)

[Supplementary Table 10: Correlation between PC1 and PC2 following outlier removal. 46](#_Toc130547567)

[Supplementary Table 11: Investigation of pleiotropy in the GWAS Catalogue and Phenoscanner databases for identified metabolite classes. 47](#_Toc130547568)

[Supplementary Table 12: Post-hoc power analysis. 49](#_Toc130547569)

#
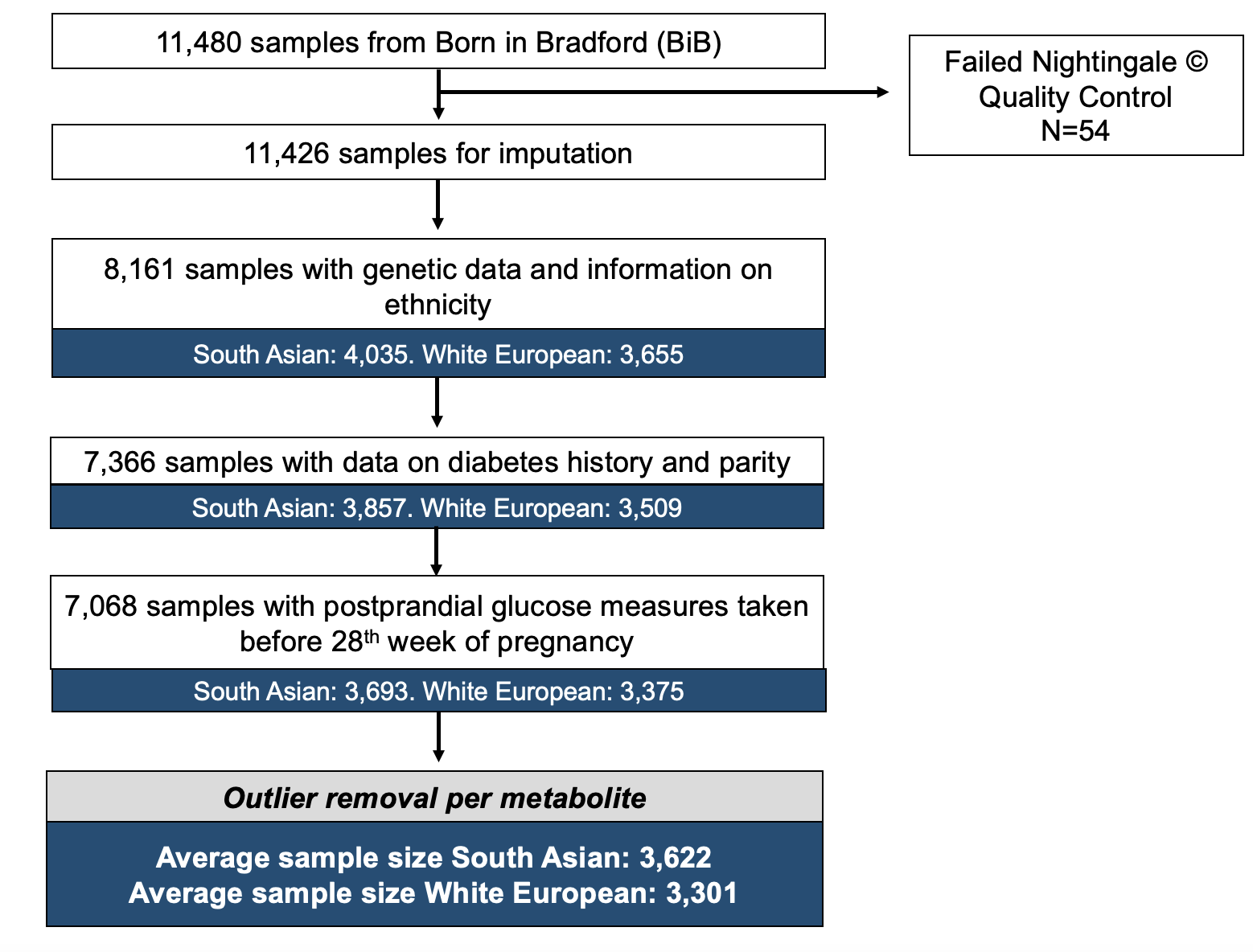
Supplementary Figure 1: Born in Bradford sample selection.

# Supplementary Figure 2: PCA to account for population stratification.


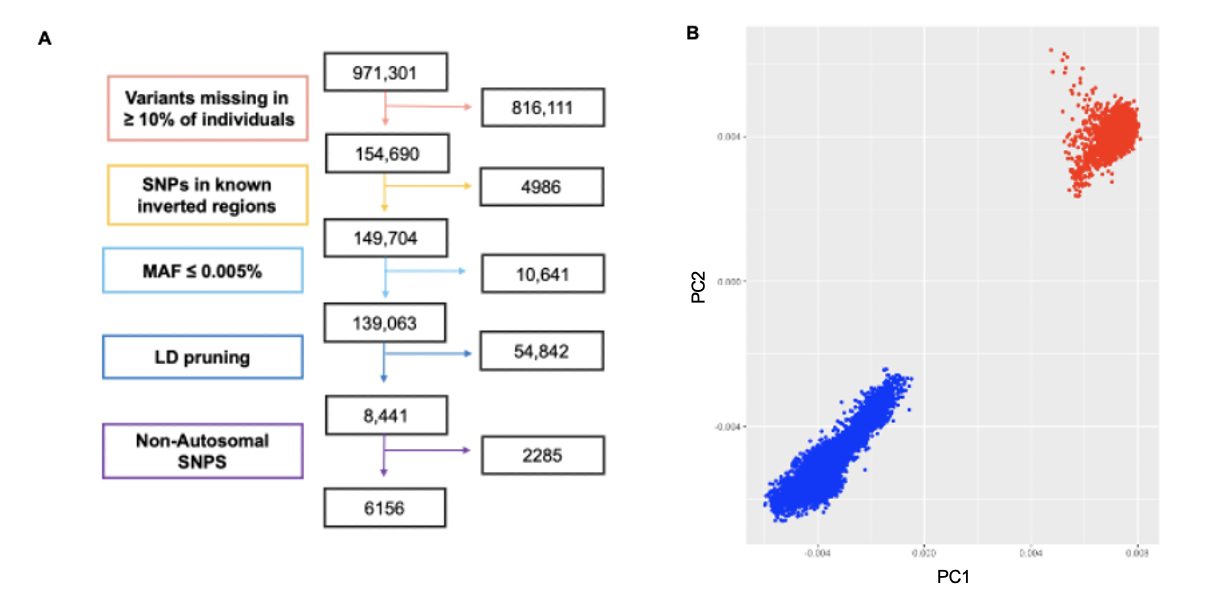


**A**: Schematic of data processing steps taken prior to PCA of genetic data. A R^2^ cut-off of 0.3 and 50 variant windows were utilised for LD pruning. =. MAF: minor allele frequency. **B**: PCA plot of BiB genotype data calculated to account for population stratification. Blue: South Asians. Red: white Europeans.

# Supplementary Figure 3: PCA comparing BiB data to all SA data from 1000G.

**
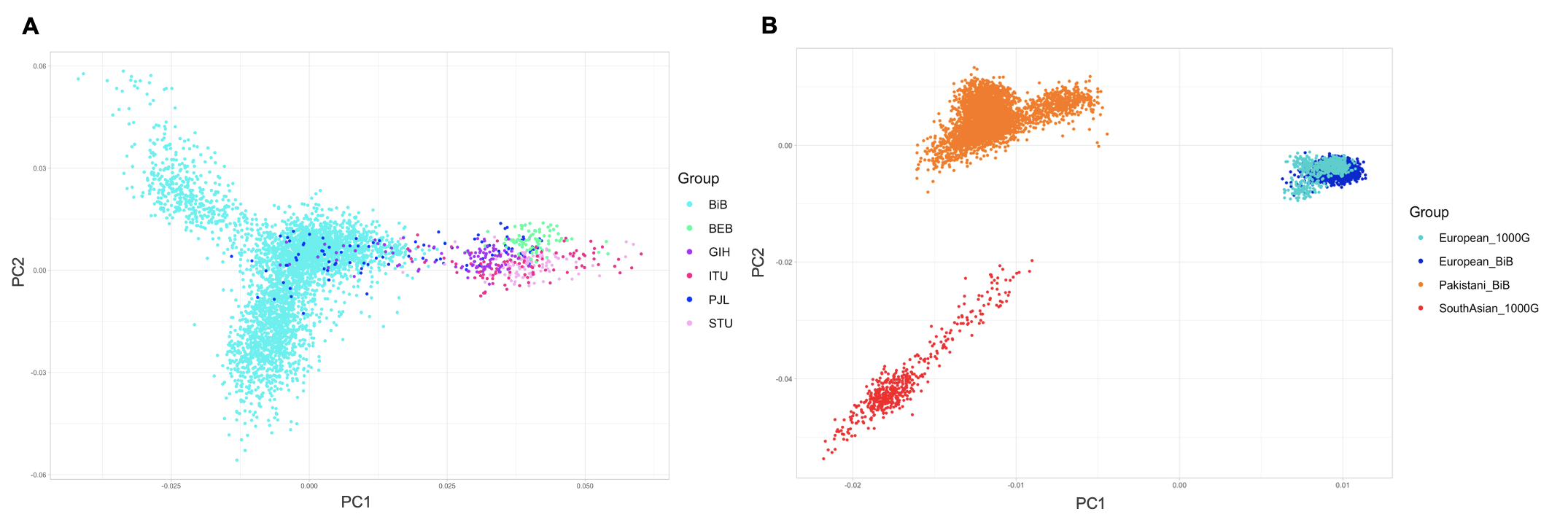
**

**A**: PCA plot of SA BiB and SA data from 1000G. BiB: Born in Bradford, BEB: Bengali in Bangladesh, GIH: Gujarati Indian from Houston, Texas. ITU: Indian Telugu in the UK. PJL: Punjabi in Lahore, Pakistan STU: Sri Lankan Tamil in the UK. **B**: PCA plot of BiB data (WE and SA) and South Asian and European data from 1000G.

# Supplementary Figure 4: PCA comparing BiB data to all data from 1000G.


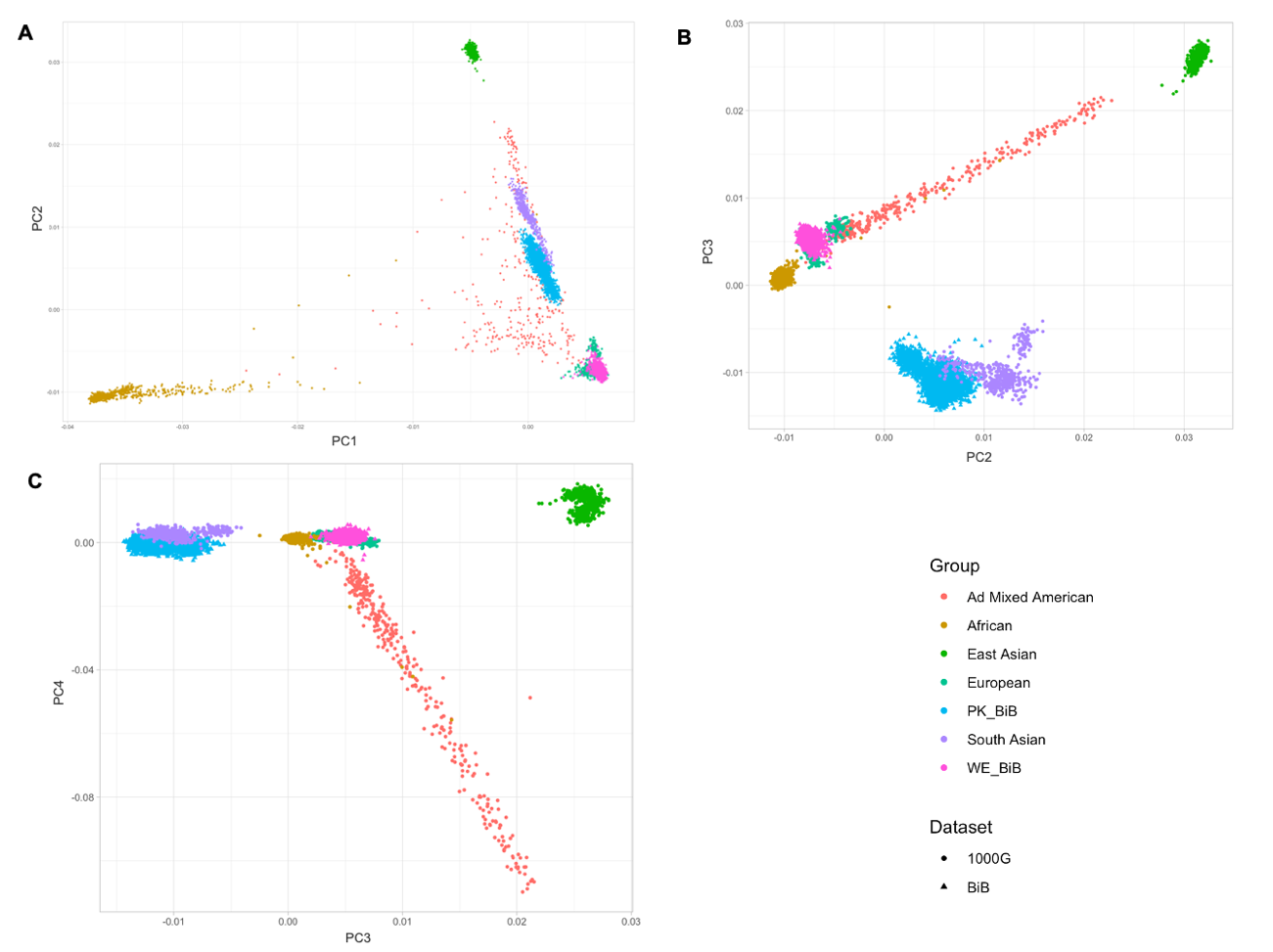


**A**: PCA plot of PC1 vs PC2. **B**: PCA plot of PC2 vs PC3. **C**: PCA plot of PC3 vs PC4. PK_BIB: Pakistani BiB sample; WE_BIB: white European BiB Sample.

# Supplementary Figure 5: PCA comparing BiB data to all data from 1000G with the Pakistani BiB and South Asian 1000G populations.


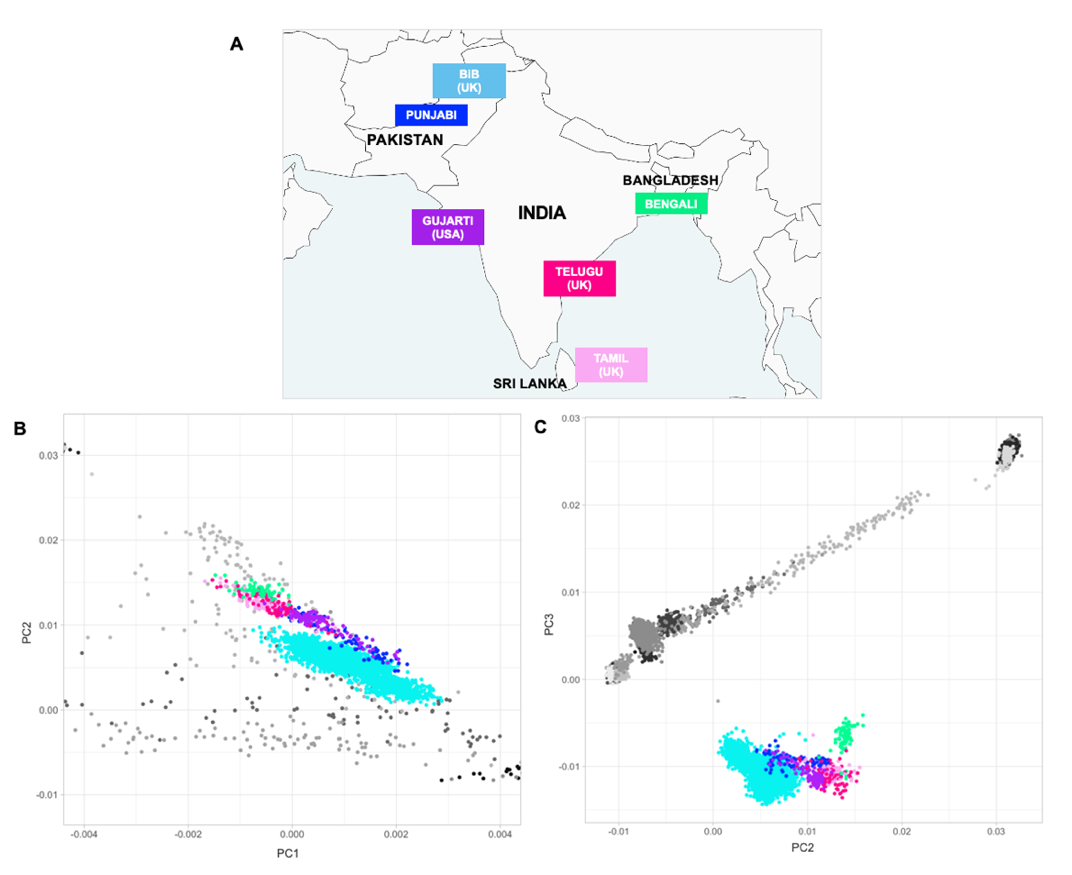


**A:** Map of the Indian subcontinent with the location of each South Asian 1000G population illustrated. Brackets represent the country in which the sample was taken from. Colours of labels illustrate data points in PCA plots **B:** PC1 vs PC2. **C:** PC2 vs PC3. Base map for panel A was obtained from the *rworld* map package in R studio.

# Supplementary Figure 6: Number of SNPs and the strength of in each instrument.

**
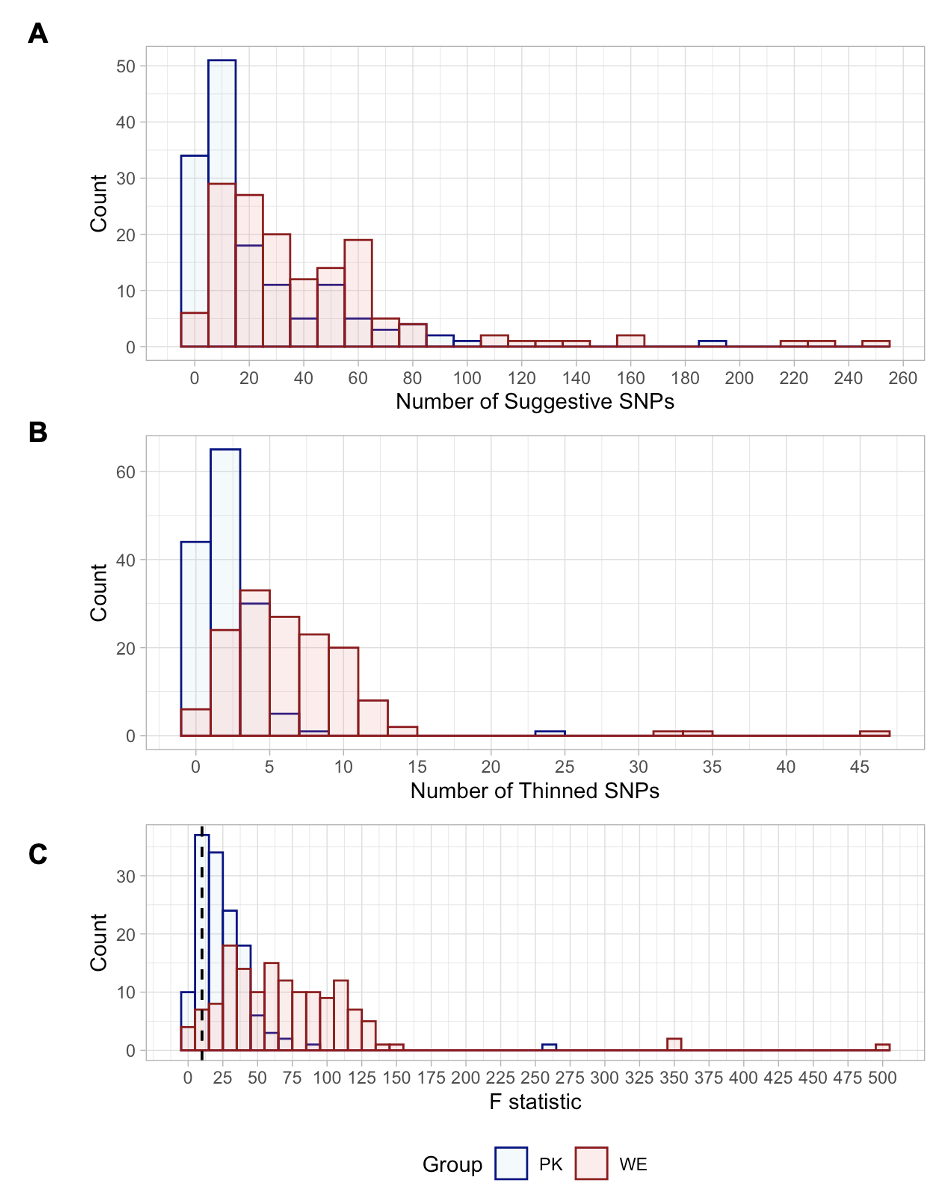
**

**A**: Histogram of number of SNPs identified for each metabolite at the suggestive level (p value 1 x 10^-5^). **B**: Histogram of the number of SNPs remaining after thinning by LD (R^2^ >0.2). **C**: Histogram of the F statistics for each instrument. Dashed line shows F statistic of 10. An F statistic < 10 is an indicator of weak instrument bias. Blue: South Asians Red: white Europeans.

# Supplementary Figure 7a: Forest plots of β values following leave-one-out analyses for white Europeans.


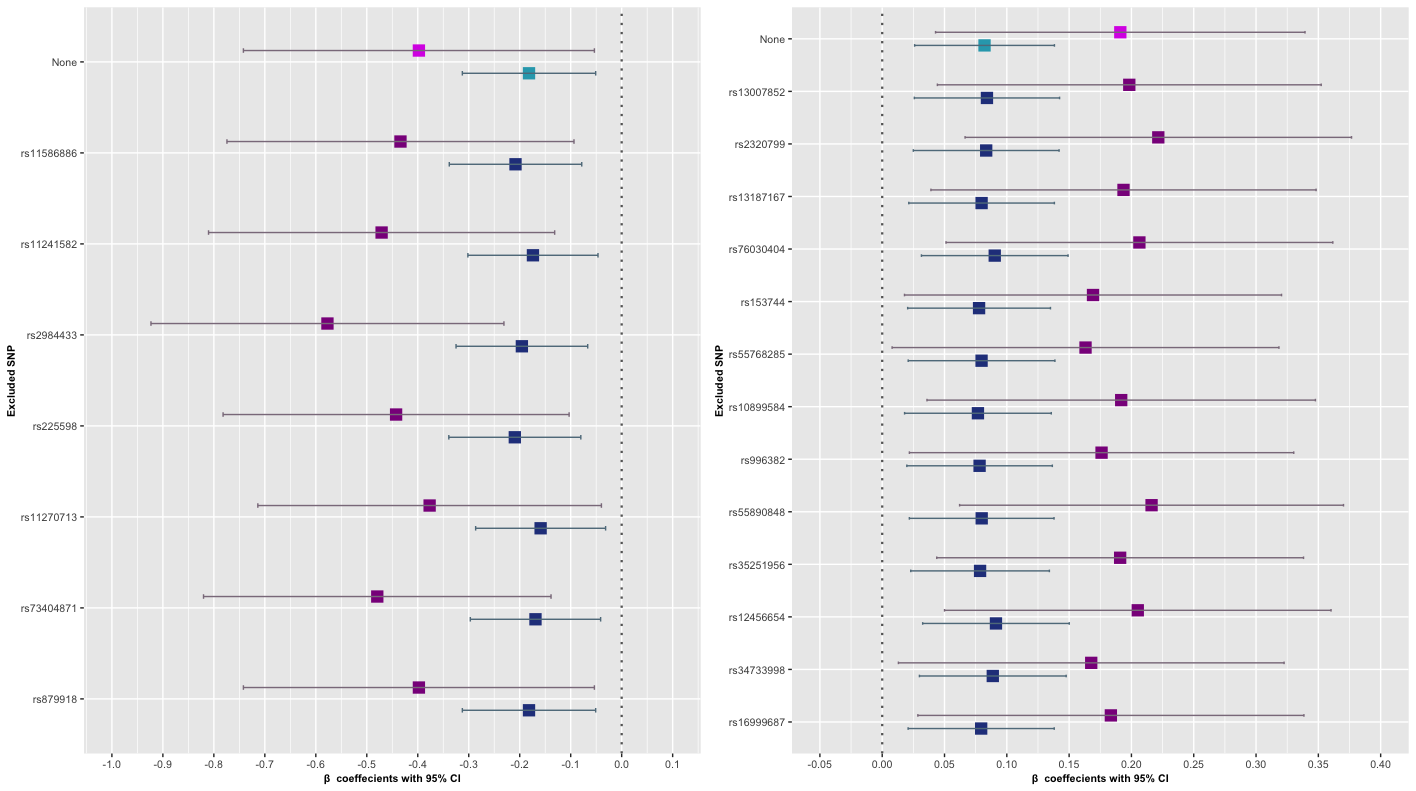


Forest plots showing β values and 95% CIs following the leave-one-out analyses of each SNP in each instrument. Dashed line represents no effect. Associations shown in purple indicating associations with 2-hour post glucose while associations shown in blue indicate associations with fasting glucose. **A**: Leucine. **B**: HDL_D.

# Supplementary Figure 7b: Forest plots of β values following leave-one-out analyses for white Europeans.

**
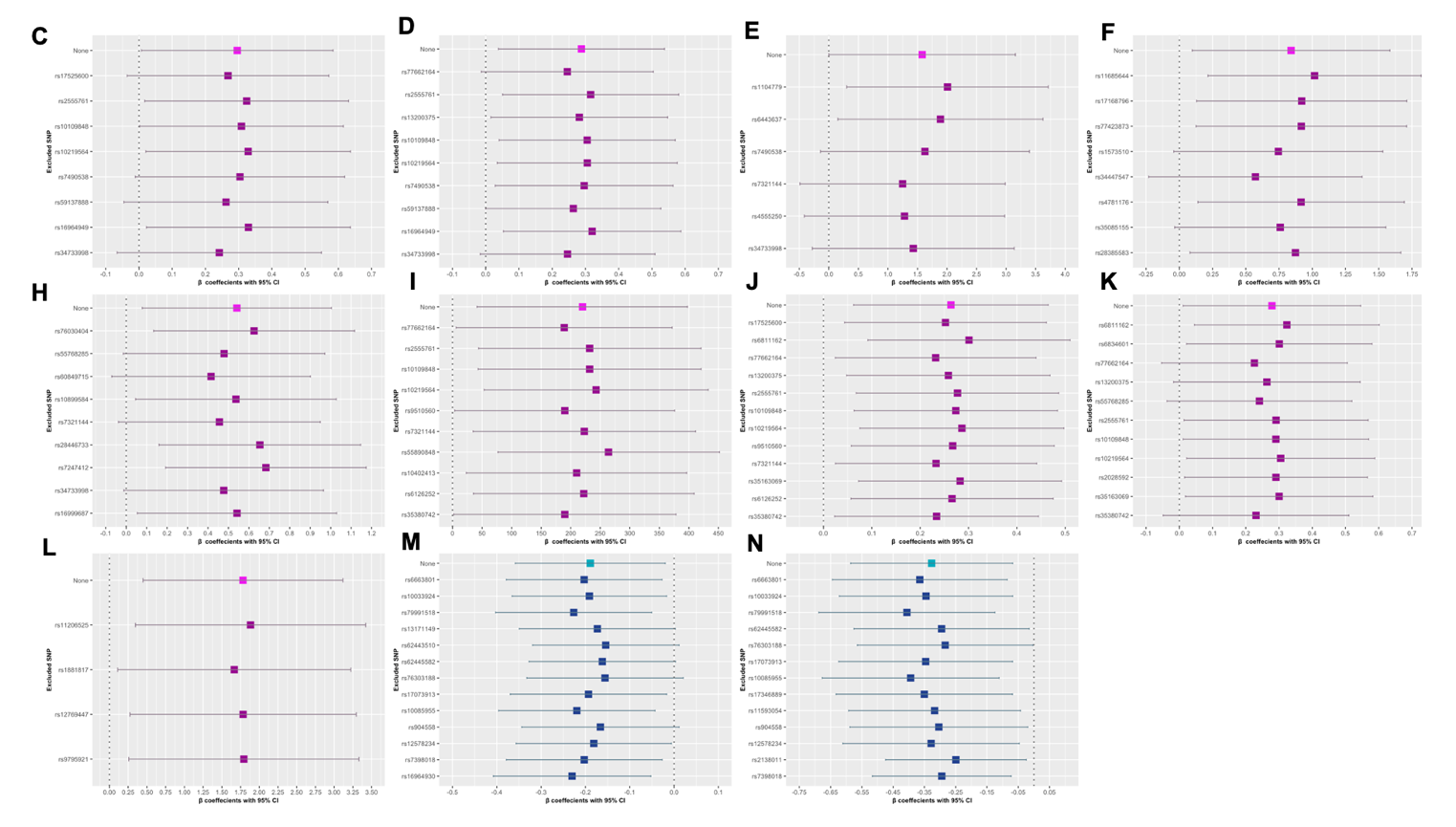
**

Forest plots showing β values and 95% CIs following the leave-one-out analyses of each SNP in each instrument. Dashed line represents no effect. Associations shown in purple indicating associations with 2-hour post glucose while associations shown in blue indicate associations with fasting glucose. **C**: HDLC. **D**: HDL2C. **E**: HDL3C. **F**: XS-VLDL-TG. **G**: XL-HDL-CE. **H**: L-HDL-P. **I**: L-HDL_L. **J**: L-HDL-C. **K**: S-HDL-CE. **L**: M-HDL-C. **M**: M-HDL-CE.

# Supplementary Figure 8: F statistics following leave-one-out analyses for identified associations in white Europeans.

#
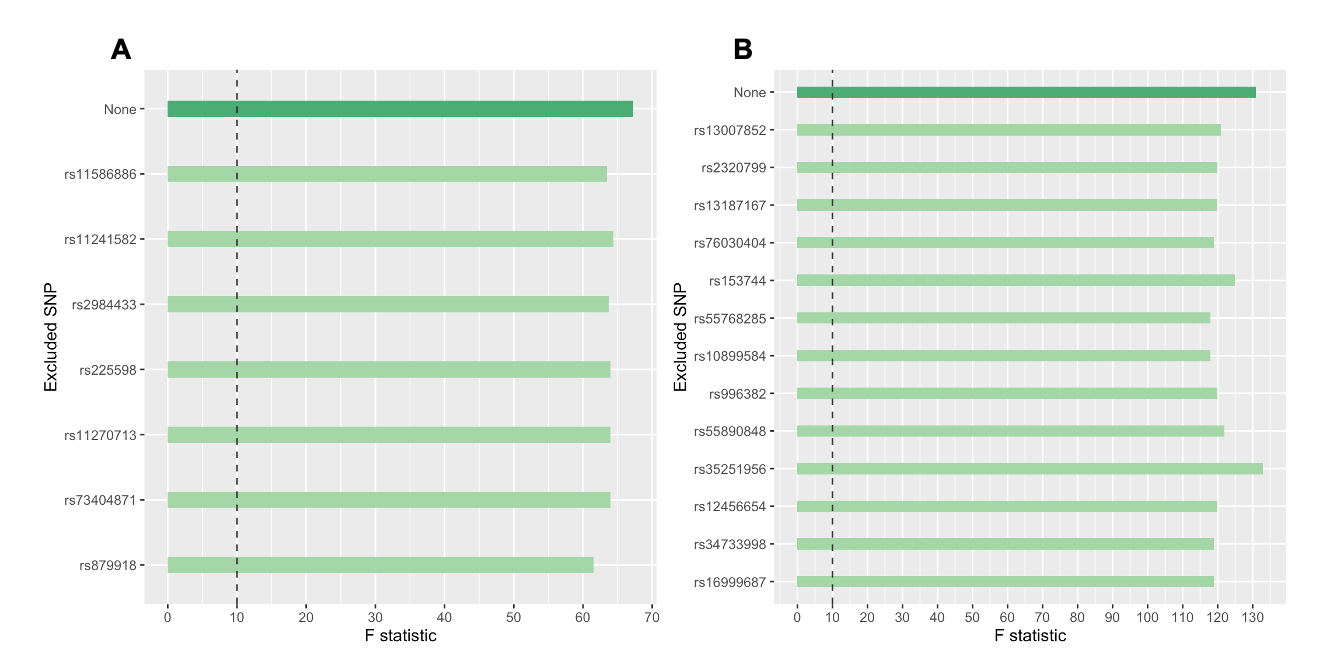


Dashed line indicates a F statistic of 10, below which an instrument is classified as weak. Associations shown in purple indicate associations with 2-hour post glucose while associations shown in blue indicate associations with fasting glucose. Green bars indicate metabolite measures associated with both fasting glucose and 2-hour post glucose. **A:** Leucine. **B**: HDL_D.

# Supplementary Figure 8 continued: F statistics following leave-one-out analyses for identified associations in white Europeans.

**
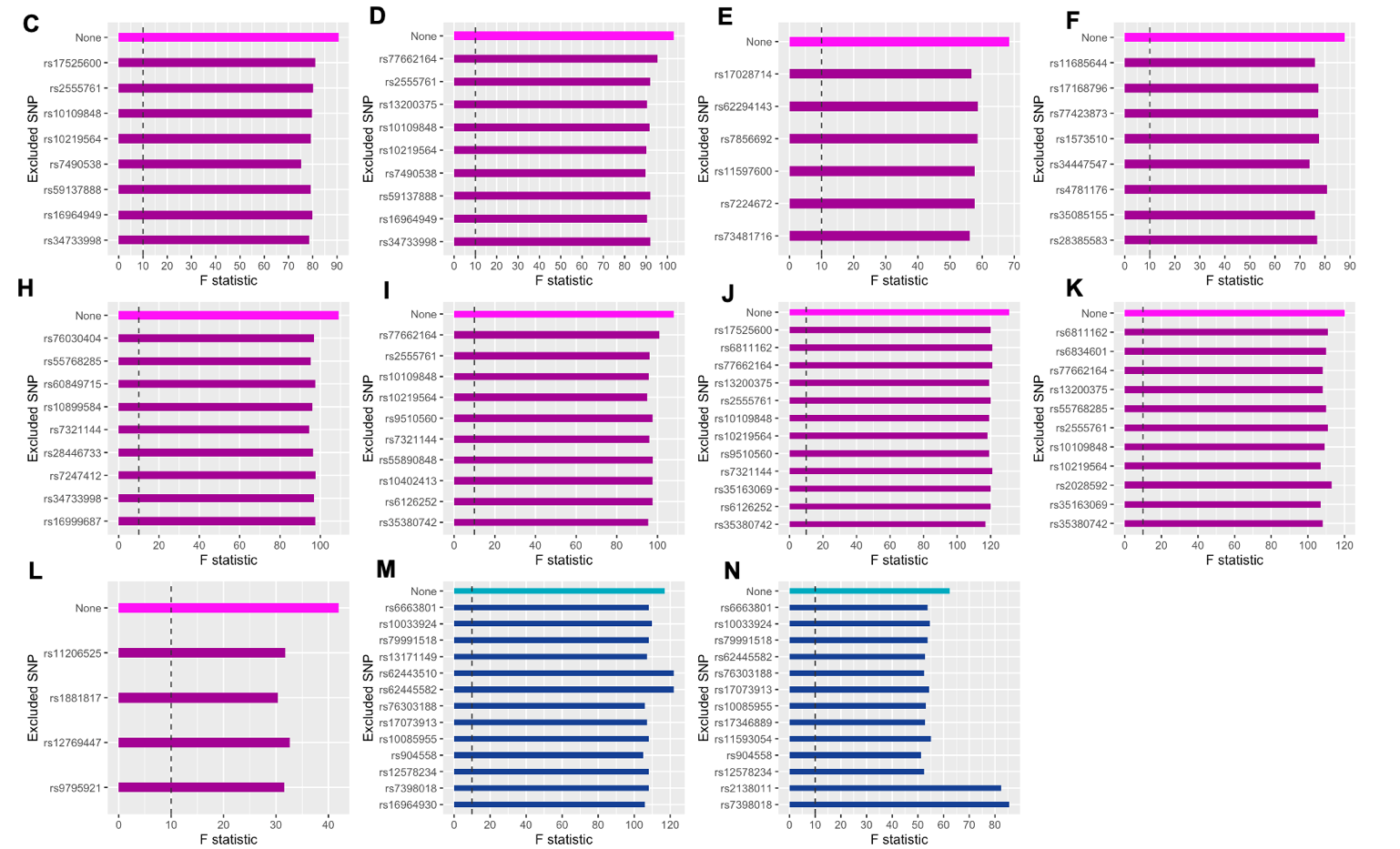
**

Dashed line indicates an F statistic of 10, below which an instrument is classified as weak. Associations shown in purple indicate associations with 2-hour post glucose while associations shown in blue indicate associations with fasting glucose. Green bars indicate metabolite measures associated with both fasting glucose and 2-hour post glucose. **C:** HDLC. **D:** HDL2C. **E:** HDL3C. **F:** XS-VLDL-TG. **G:** XL-HDL-CE. **H:** L-HDL-P. **I:** L-HDL_L. **J:** L-HDL-C. **K:** S-HDL-CE. **L:** M-HDL-C. **M:** M-HDL-CE

# Supplementary Figure 9: Forest plots of β values following leave-one-out analyses for identified associations in South Asians.

#
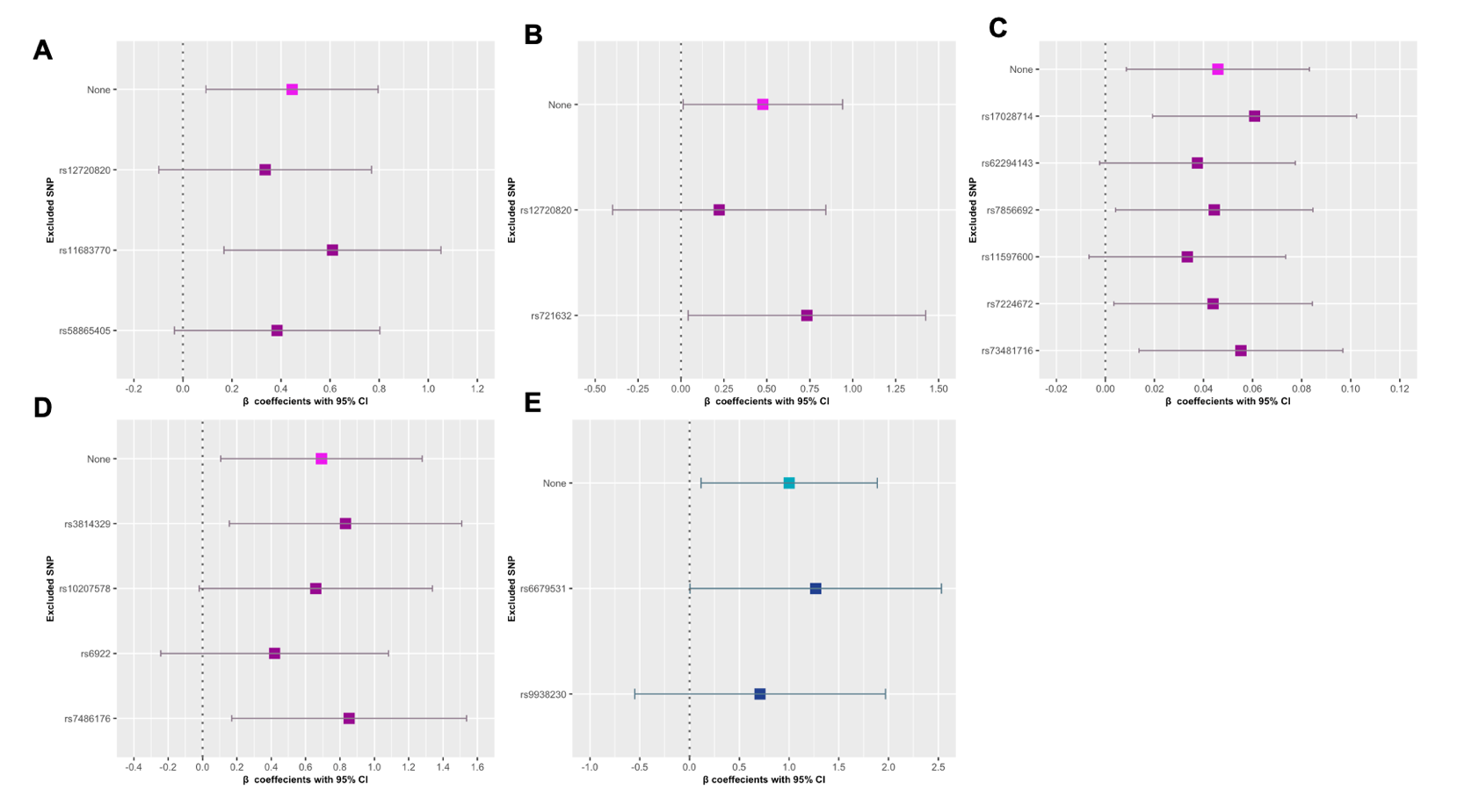


# Supplementary Figure 10: F statistics following leave-one- out analyses for identified associations in South Asians.

**
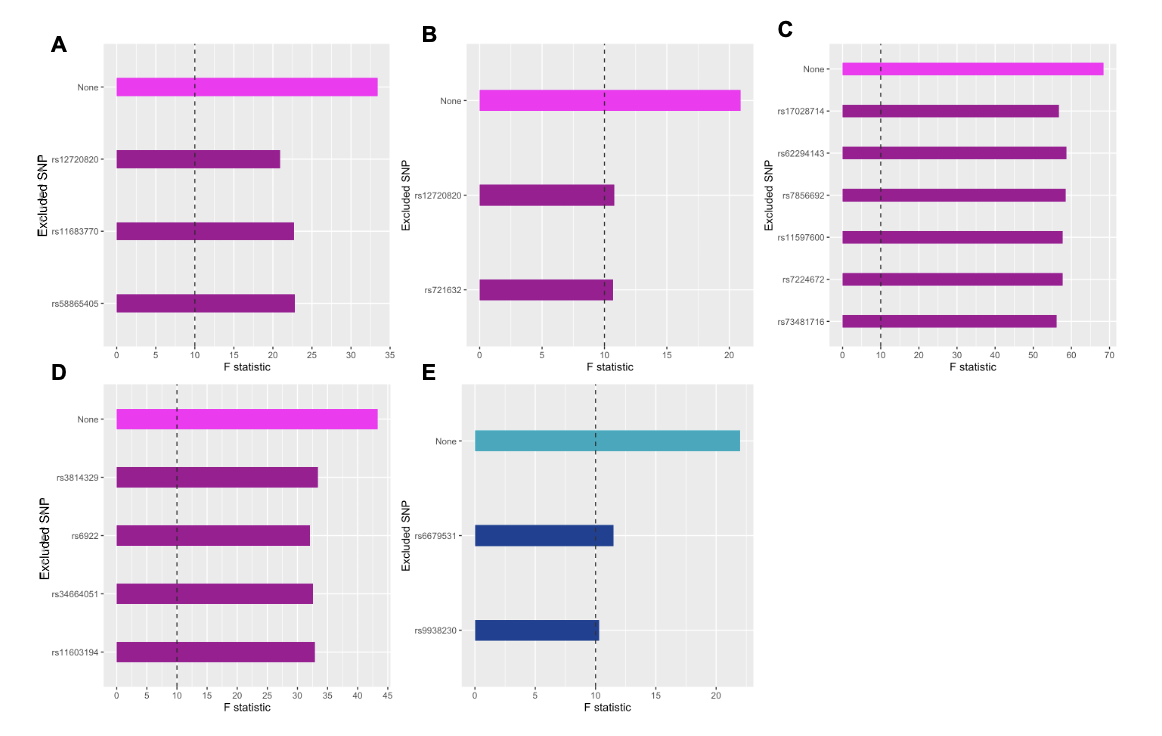
**

Dashed line indicates an F statistic of 10, below which an instrument is classified as weak Associations shown in purple indicate associations with 2-hour post glucose while associations shown in blue indicate associations with fasting glucose. **A:** FAw6. **B:** LA. **C:** M-VLDL-L. **D:** L-HDL-PL. **E:** S-HDL-C.

# Supplementary Figure 11: Overlap of SNPs identified by class in white Europeans.

**
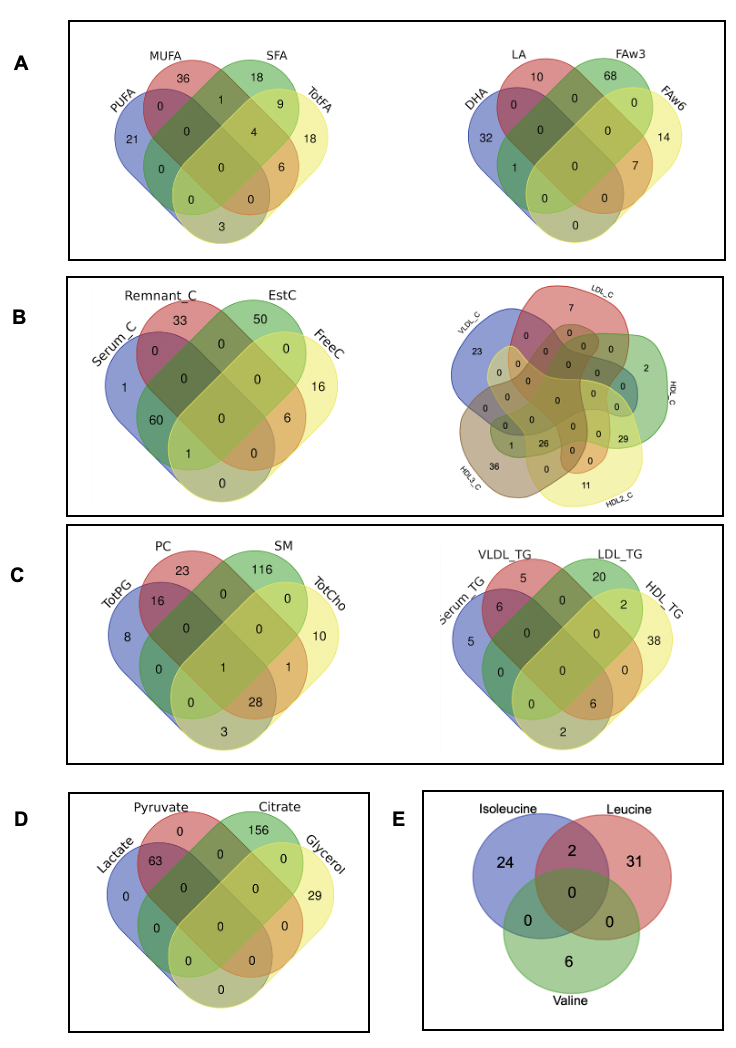
**

Venn diagram showing the overlap between suggestive (p value 1 x 10^-5^) SNPs in WEs. SNPs in the apolipoprotein, lipoprotein density, ketone Bodies and fluid Balance/ inflammation, aromatic amino acids and non-branched amino acids were not found to overlap in WEs. Classes with ≥5 metabolites have been split for clarity. **A**: Fatty Acids. **B**: Cholesterols. **C**: Glycerides and Phospholipids. **D**: Glycolysis related metabolites. **E**: Branched amino acids.

# Supplementary Figure 12: Overlap of SNPs identified by class in South Asians.


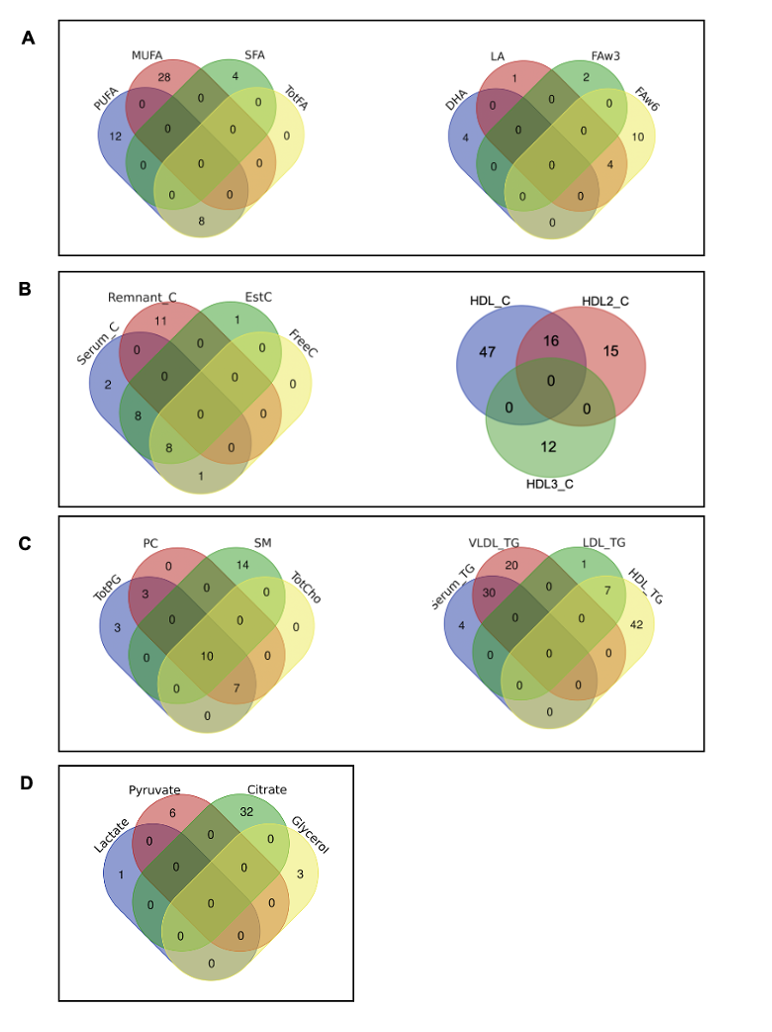


Venn diagram highlighting the overlap between suggestive (p value 1 x 10^-5^) SNPs in SAs. SNPs in the apolipoprotein, lipoprotein density, ketone Bodies and fluid Balance/ inflammation, and amino acids were not found to overlap in WEs. Classes with ≥5 metabolites have been split for clarity. **A**: Fatty Acids. **B**: Cholesterols. **C**: Glycerides and Phospholipids. **D**: Glycolysis related metabolites

# Supplementary Figure 13: Number of SNPs and strength of each instrument in the analysis of each metabolite class.


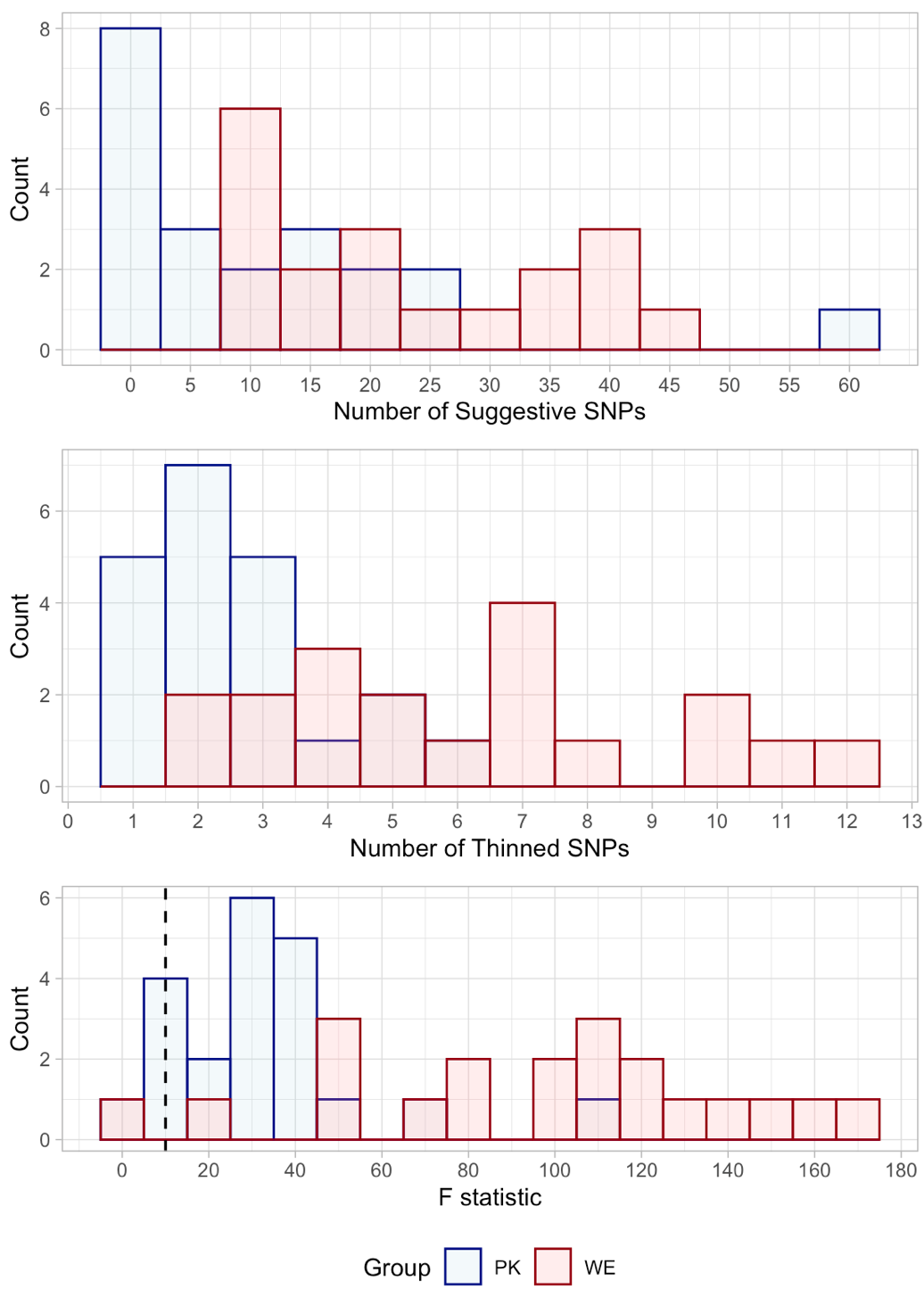


**A:** Histogram of number of SNPs identified for each metabolite class at the suggestive level (p≤ 1 x 10^-5^). **B:** Histogram of the number of SNPs remaining after thinning by LD (R^2^>0.2).**C**: Histogram of the F statistics for each instrument. Dashed line shows an F statistic of 10. An F statistic < 10 is an indicator of weak instrument bias. Blue: South Asians. Red: white Europeans.

# Supplementary Figure 14: Forest plots of β values following leave-one-out analyses for identified associations in the analysis of metabolite class.

**
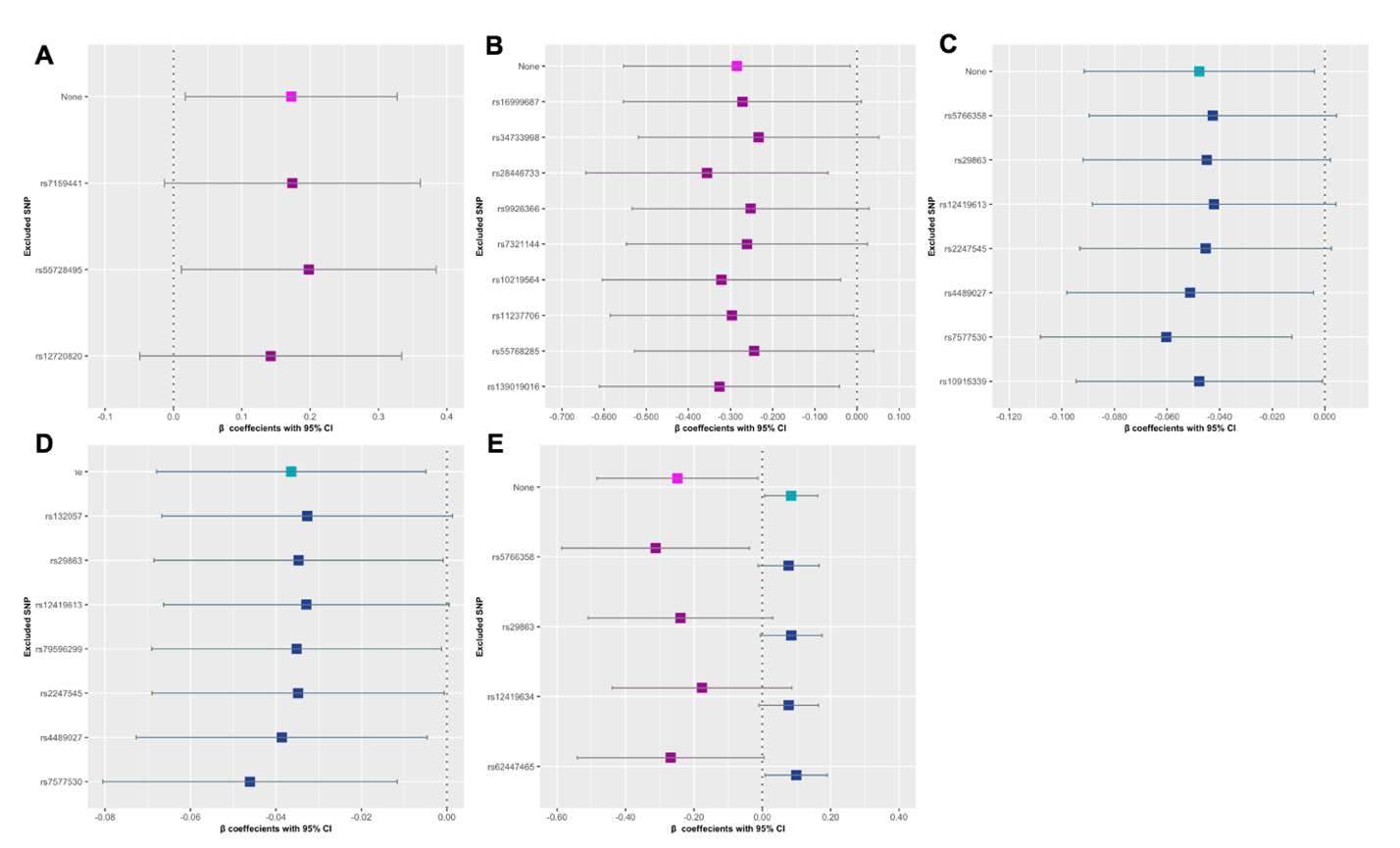
**

Forest plots showing β values and 95% CIs following the leave-one-out analyses of each SNP in each instrument. Dashed line represents no effect. Associations shown in purple indicate associations with 2-hour post glucose while associations shown in blue indicate associations with fasting glucose. **A:** Fatty acid class, SAs. **B:** XL-HDL class, WEs. **C:** M-LDL class, WEs. **D:** All LDL class, WEs. **E:** S-LDL class, WEs.

# Supplementary Figure 15: F statistics following leave-one-out analyses for identified associations in the analysis of metabolite classes.

#
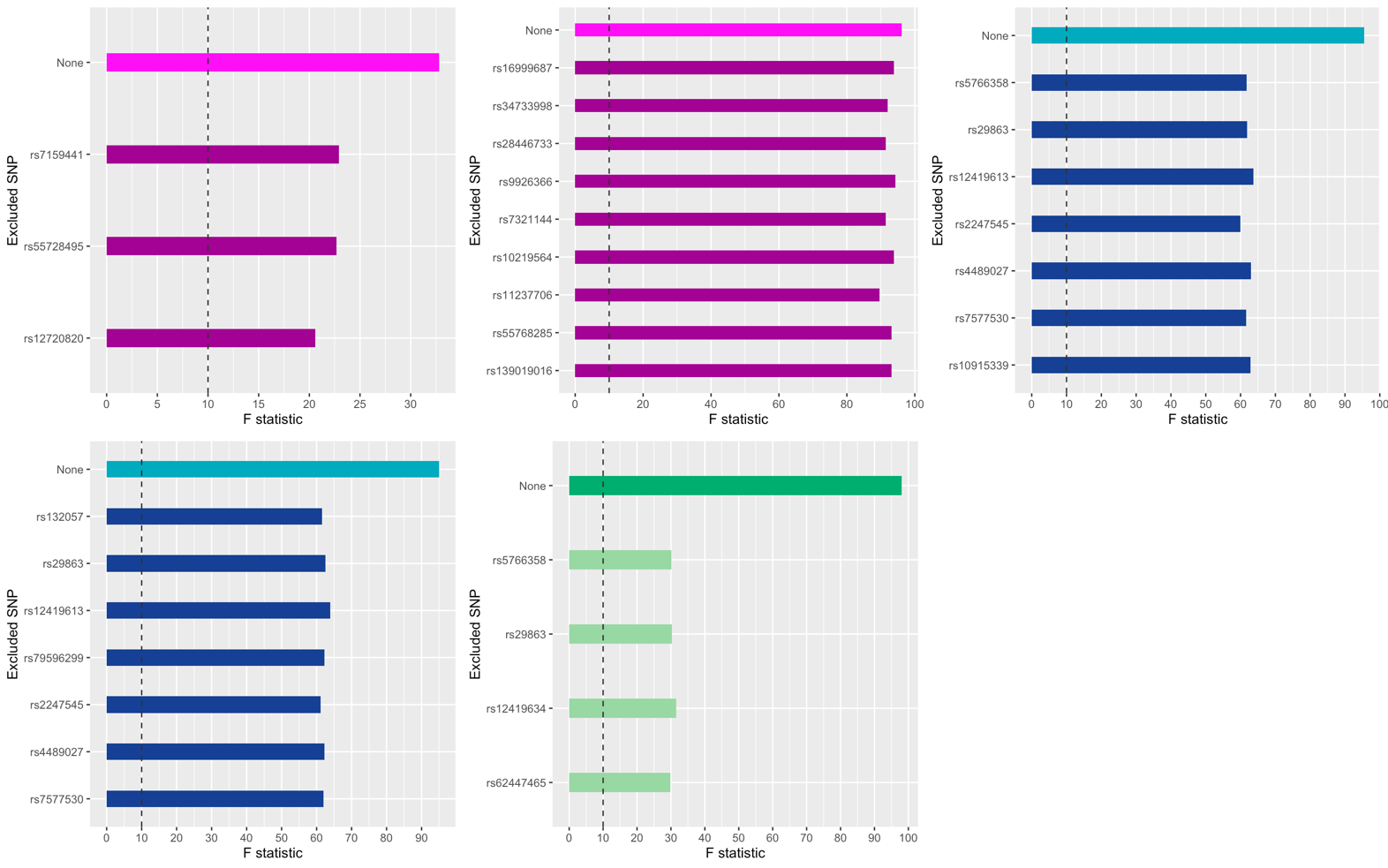


Dashed line indicates an F statistic of 10, below which an instrument is classified as weak. Associations shown in purple indicating associations with 2-hour post glucose while associations shown in blue indicate associations with fasting glucose. Green bars indicate metabolite measures associated with both fasting glucose and 2-hour post glucose. **A:** Fatty acid class, SAs. **B:** XL-HDL class, WEs. **C:** M-LDL class, WEs. **D:** All LDL class, WEs. **E:** S-LDL class, WEs.

# Supplementary Table 1: List of metabolite values included within this study.

| Metabolite Class | Metabolite Code | Full Name (Unit) |
| --- | --- | --- |
| XXL-VLDL | XXL-VLDL-P | Concentration of XXL-VLDL (mol/l) |
|  | XXL-VLDL-L | Total lipids in XXL-VLDL (mmol/l) |
|  | XXL-VLDL-PL | Phospholipids in XXL-VLDL (mmol/l) |
|  | XXL-VLDL-C | Total Cholesterol in XXL-VLDL (mmol/l) |
|  | XXL-VLDL-CE | Cholesterol esters in XXL-VLDL (mmol/l) |
|  | XXL-VLDL-FC | Free cholesterol in XXL-VLDL (mmol/l) |
|  | XXL-VLDL-TG | Triglycerides in XXL-VLDL (mmol/l) |
| XL-VLDL | XL-VLDL-P | Concentration of XL-VLDL (mol/l) |
|  | XL-VLDL-L | Total lipids in XL-VLDL (mmol/l) |
|  | XL-VLDL-PL | Phospholipids in XL-VLDL (mmol/l) |
|  | XL-VLDL-C | Total Cholesterol in XL-VLDL (mmol/l) |
|  | XL-VLDL-CE | Cholesterol esters in XL-VLDL (mmol/l) |
|  | XL-VLDL-FC | Free cholesterol in XL-VLDL (mmol/l) |
|  | XL-VLDL-TG | Triglycerides in XL-VLDL (mmol/l) |
| L-VLDL | L-VLDL-P | Concentration of L-VLDL (mol/l) |
|  | L-VLDL-L | Total lipids in L-VLDL (mmol/l) |
|  | L-VLDL-PL | Phospholipids in L-VLDL (mmol/l) |
|  | L-VLDL-C | Total Cholesterol in L-VLDL (mmol/l) |
|  | L-VLDL-CE | Cholesterol esters in L-VLDL (mmol/l) |
|  | L-VLDL-FC | Free cholesterol in L-VLDL (mmol/l) |
|  | L-VLDL-TG | Triglycerides in L-VLDL (mmol/l) |
| M-VLDL | M-VLDL-P | Concentration of M-VLDL (mol/l) |
|  | M-VLDL-L | Total lipids in M-VLDL (mmol/l) |
|  | M-VLDL-PL | Phospholipids in M-VLDL (mmol/l) |
|  | M-VLDL-C | Total Cholesterol in M-VLDL (mmol/l) |
|  | M-VLDL-CE | Cholesterol esters in M-VLDL (mmol/l) |
|  | M-VLDL-FC | Free cholesterol in M-VLDL (mmol/l) |
|  | M-VLDL-TG | Triglycerides in M-VLDL (mmol/l) |
| S-VLDL | S-VLDL-P | Concentration of S-VLDL (mol/l) |
|  | S-VLDL-L | Total lipids in S-VLDL (mmol/l) |
|  | S-VLDL-PL | Phospholipids in S-VLDL (mmol/l) |
|  | S-VLDL-C | Total Cholesterol in S-VLDL (mmol/l) |
|  | S-VLDL-CE | Cholesterol esters in S-VLDL (mmol/l) |
|  | S-VLDL-FC | Free cholesterol in S-VLDL (mmol/l) |
|  | S-VLDL-TG | Triglycerides in S-VLDL (mmol/l) |
| XS-VLDL | XS-VLDL-P | Concentration of XS-VLDL (mol/l) |
|  | XS-VLDL-L | Total lipids in XS-VLDL (mmol/l) |
|  | XS-VLDL-PL | Phospholipids in XS-VLDL (mmol/l) |
|  | XS-VLDL-C | Total Cholesterol in XS-VLDL (mmol/l) |
|  | XS-VLDL-CE | Cholesterol esters in XS-VLDL (mmol/l) |
|  | XS-VLDL-FC | Free cholesterol in XS-VLDL (mmol/l) |
|  | XS-VLDL-TG | Triglycerides in XS-VLDL (mmol/l) |
| IDL | IDL-P | Concentration of IDL-P (mol/l) |
|  | IDL-L | Total lipids in IDL-L (mmol/l) |
|  | IDL-PL | Phospholipids in IDL-PL (mmol/l) |
|  | IDL-C | Total Cholesterol in IDL-C (mmol/l) |
|  | IDL-CE | Cholesterol esters in IDL-CE (mmol/l) |
|  | IDL-FC | Free cholesterol in IDL-FC (mmol/l) |
|  | IDL-TG | Triglycerides in IDL-TG (mmol/l) |
| L-LDL | L-LDL-P | Concentration of L-LDL-P (mol/l) |
|  | L-LDL-L | Total lipids in L-LDL-L (mmol/l) |
|  | L-LDL-PL | Phospholipids in L-LDL-PL (mmol/l) |
|  | L-LDL-C | Total Cholesterol in L-LDL-C (mmol/l) |
|  | L-LDL-CE | Cholesterol esters in L-LDL-CE (mmol/l) |
|  | L-LDL-FC | Free cholesterol in L-LDL-FC (mmol/l) |
|  | L-LDL-TG | Triglycerides in L-LDL-TG (mmol/l) |
| M-LDL | M-LDL-P | Concentration of M-LDL-P (mol/l) |
|  | M-LDL-L | Total lipids in M-LDL-L (mmol/l) |
|  | M-LDL-PL | Phospholipids in M-LDL-PL (mmol/l) |
|  | M-LDL-C | Total Cholesterol in M-LDL-C (mmol/l) |
|  | M-LDL-CE | Cholesterol esters in M-LDL-CE (mmol/l) |
|  | M-LDL-FC | Free cholesterol in M-LDL-FC (mmol/l) |
|  | M-LDL-TG | Triglycerides in M-LDL-TG (mmol/l) |
| S-LDL | S-LDL-P | Concentration of S-LDL-P (mol/l) |
|  | S-LDL-L | Total lipids in S-LDL-L (mmol/l) |
|  | S-LDL-PL | Phospholipids in S-LDL-PL (mmol/l) |
|  | S-LDL-C | Total Cholesterol in S-LDL-C (mmol/l) |
|  | S-LDL-CE | Cholesterol esters in S-LDL-CE (mmol/l) |
|  | S-LDL-FC | Free cholesterol in S-LDL-FC (mmol/l) |
|  | S-LDL-TG | Triglycerides in S-LDL-TG (mmol/l) |
| XL-HDL | XL-HDL-P | Concentration of XL-HDL-P (mol/l) |
|  | XL-HDL-L | Total lipids in XL-HDL-L (mmol/l) |
|  | XL-HDL-PL | Phospholipids in XL-HDL-PL (mmol/l) |
|  | XL-HDL-C | Total Cholesterol in XL-HDL-C (mmol/l) |
|  | XL-HDL-CE | Cholesterol esters in XL-HDL-CE (mmol/l) |
|  | XL-HDL-FC | Free cholesterol in XL-HDL-FC (mmol/l) |
|  | XL-HDL-TG | Triglycerides in XL-HDL-TG (mmol/l) |
| L-HDL | L-HDL-P | Concentration of L-HDL-P (mol/l) |
|  | L-HDL-L | Total lipids in L-HDL-L (mmol/l) |
|  | L-HDL-PL | Phospholipids in L-HDL-PL (mmol/l) |
|  | L-HDL-C | Total Cholesterol in L-HDL-C (mmol/l) |
|  | L-HDL-CE | Cholesterol esters in L-HDL-CE (mmol/l) |
|  | L-HDL-FC | Free cholesterol in L-HDL-FC (mmol/l) |
|  | L-HDL-TG | Triglycerides in L-HDL-TG (mmol/l) |
| M-HDL | M-HDL-P | Concentration of M-HDL-P (mol/l) |
|  | M-HDL-L | Total lipids in M-HDL-L (mmol/l) |
|  | M-HDL-PL | Phospholipids in M-HDL-PL (mmol/l) |
|  | M-HDL-C | Total Cholesterol in M-HDL-C (mmol/l) |
|  | M-HDL-CE | Cholesterol esters in M-HDL-CE (mmol/l) |
|  | M-HDL-FC | Free cholesterol in M-HDL-FC (mmol/l) |
|  | M-HDL-TG | Triglycerides in M-HDL-TG (mmol/l) |
| S-HDL | S-HDL-P | Concentration of S-HDL-P (mol/l) |
|  | S-HDL-L | Total lipids in S-HDL-L (mmol/l) |
|  | S-HDL-PL | Phospholipids in S-HDL-PL (mmol/l) |
|  | S-HDL-C | Total Cholesterol in S-HDL-C (mmol/l) |
|  | S-HDL-CE | Cholesterol esters in S-HDL-CE (mmol/l) |
|  | S-HDL-FC | Free cholesterol in S-HDL-FC (mmol/l) |
|  | S-HDL-TG | Triglycerides in S-HDL-TG (mmol/l) |
| Lipoprotien Density | VLDL_D | Mean diameter of VLDL (nm) |
|  | LDL_D | Mean diameter of LDL (nm) |
|  | HDL_D | Mean diameter of HDL (nm) |
| Cholesterols | Serum_C | Total serum cholesterol (mmol/l) |
|  | VLDL_C | VLDL cholesterol (mmol/l) |
|  | Remnant_C | Remnant cholesterol* (mmol/l) |
|  | LDL_C | LDL cholesterol (mmol/l) |
|  | HDL_C | HDL cholesterol (mmol/l) |
|  | HDL2_C | HDL2 cholesterol (mmol/l) |
|  | HDL3_C | HDL3 cholesterol (mmol/l) |
|  | EstC | Total esterified cholesterol (mmol/l) |
|  | FreeC | Free Cholesterol (mmol/l) |
| Glycerides and Phospholipids | Serum_TG | Total serum triglycerides (mmol/l) |
|  | VLDL-TG | VLDL triglycerides (mmol/l) |
|  | LDL-TG | LDL triglycerides (mmol/l) |
|  | HDL-TG | HDL triglycerides (mmol/l) |
|  | TotPG | Total phosphoglycerides (mmol/l) |
|  | PC | Phosphatidylcholine* (mmol/l) |
|  | SM | Sphingomyelins (mmol/l) |
|  | TotCho | Total cholines (mmol/l) |
| Apolipoproteins | ApoA1 | Apolipoprotein A1 (g/l) |
|  | ApoB | Apolipoprotein B (g/l) |
| Fatty Acids | TotFA | Total fatty acids (mmol/l) |
|  | DHA | 22:6, docosahexaenoic acid (mmol/l) |
|  | LA | 18:2, linoleic acid (mmol/l) |
|  | FAw3 | Omega-3 fatty acids (mmol/l) |
|  | FAw6 | Omega-6 fatty acids (mmol/l) |
|  | PUFA | Polyunsaturated fatty acids |
|  | MUFA | Monounsaturated fatty acids ** (mmol/l) |
|  | SFA | Saturated fatty acids (mmol/l) |
| Glycolysis Related Metabolites | Lac | Lactate (mmol/l) |
|  | Pyr | Pyruvate (mmol/l) |
|  | Cit | Citrate (mmol/l) |
|  | Glol | Glycerol (mmol/l) |
| Amino Acids | Ala | Alanine (mmol/l) |
|  | Gln | Glycine (mmol/l) |
|  | Gly | Glycerol (mmol/l) |
|  | His | Histidine (mmol/l) |
|  | Ile | Isoleucine (mmol/l) |
|  | Leu | Leucine (mmol/l) |
|  | Val | Valine (mmol/l) |
|  | Phe | Phenylalanine (mmol/l) |
|  | Tyr | Tyrosine (mmol/l) |
| Ketone Bodies | Ace | Acetate (mmol/l) |
|  | bOHBut | 3-hydroxybutyrate (mmol/l) |
| Fluid Balance and Inflammation | Crea | Creatine (mmol/l) |
|  | Alb | Albumin (signal area) |
|  | Gp | Glycoprotein acetyls (mmol/l) |

# Supplementary Table 2: Proportion of outliers identified in each ethnicity.

|  | South Asian | | White European | |
| --- | --- | --- | --- | --- |
| Metabolite | **Number kept** | **% Lost** | **Number kept** | **% Lost** |
| XXL-VLDL-P | 3586 | 2.90 | 3247 | 3.79 |
| XXL-VLDL-L | 3589 | 2.82 | 3250 | 3.70 |
| XXL-VLDL-PL | 3592 | 2.73 | 3253 | 3.61 |
| XXL-VLDL-C | 3605 | 2.38 | 3270 | 3.11 |
| XXL-VLDL-CE | 3615 | 2.11 | 3276 | 2.93 |
| XXL-VLDL-FC | 3591 | 2.76 | 3264 | 3.29 |
| XXL-VLDL-TG | 3584 | 2.95 | 3241 | 3.97 |
| XL-VLDL-P | 3585 | 2.92 | 3261 | 3.38 |
| XL-VLDL-L | 3587 | 2.87 | 3261 | 3.38 |
| XL-VLDL-PL | 3587 | 2.87 | 3255 | 3.56 |
| XL-VLDL-C | 3599 | 2.55 | 3259 | 3.44 |
| XL-VLDL-CE | 3600 | 2.52 | 3256 | 3.53 |
| XL-VLDL-FC | 3595 | 2.65 | 3258 | 3.47 |
| XL-VLDL-TG | 3587 | 2.87 | 3262 | 3.35 |
| L-VLDL-P | 3588 | 2.84 | 3264 | 3.29 |
| L-VLDL-L | 3592 | 2.73 | 3265 | 3.26 |
| L-VLDL-PL | 3596 | 2.63 | 3266 | 3.23 |
| L-VLDL-C | 3590 | 2.79 | 3257 | 3.50 |
| L-VLDL-CE | 3607 | 2.33 | 3256 | 3.53 |
| L-VLDL-FC | 3593 | 2.71 | 3265 | 3.26 |
| L-VLDL-TG | 3588 | 2.84 | 3262 | 3.35 |
| M-VLDL-P | 3601 | 2.49 | 3266 | 3.23 |
| M-VLDL-L | 3603 | 2.44 | 3261 | 3.38 |
| M-VLDL-PL | 3610 | 2.25 | 3268 | 3.17 |
| M-VLDL-C | 3614 | 2.14 | 3271 | 3.08 |
| M-VLDL-CE | 3616 | 2.09 | 3289 | 2.55 |
| M-VLDL-FC | 3608 | 2.30 | 3266 | 3.23 |
| M-VLDL-TG | 3589 | 2.82 | 3268 | 3.17 |
| S-VLDL-P | 3617 | 2.06 | 3295 | 2.37 |
| S-VLDL-L | 3615 | 2.11 | 3291 | 2.49 |
| S-VLDL-PL | 3627 | 1.79 | 3301 | 2.19 |
| S-VLDL-C | 3627 | 1.79 | 3303 | 2.13 |
| S-VLDL-CE | 3627 | 1.79 | 3309 | 1.96 |
| S-VLDL-FC | 3617 | 2.06 | 3300 | 2.22 |
| S-VLDL-TG | 3606 | 2.36 | 3271 | 3.08 |
| XS-VLDL-P | 3623 | 1.90 | 3316 | 1.75 |
| XS-VLDL-L | 3627 | 1.79 | 3315 | 1.78 |
| XS-VLDL-PL | 3627 | 1.79 | 3311 | 1.90 |
| XS-VLDL-C | 3632 | 1.65 | 3305 | 2.07 |
| XS-VLDL-CE | 3634 | 1.60 | 3308 | 1.99 |
| XS-VLDL-FC | 3626 | 1.81 | 3308 | 1.99 |
| XS-VLDL-TG | 3614 | 2.14 | 3295 | 2.37 |
| IDL-P | 3628 | 1.76 | 3312 | 1.87 |
| IDL-L | 3629 | 1.73 | 3310 | 1.93 |
| IDL-PL | 3628 | 1.76 | 3307 | 2.01 |
| IDL-C | 3628 | 1.76 | 3311 | 1.90 |
| IDL-CE | 3630 | 1.71 | 3310 | 1.93 |
| IDL-FC | 3624 | 1.87 | 3318 | 1.69 |
| IDL-TG | 3619 | 2.00 | 3303 | 2.13 |
| L-LDL-P | 3630 | 1.71 | 3307 | 2.01 |
| L-LDL-L | 3630 | 1.71 | 3308 | 1.99 |
| L-LDL-PL | 3632 | 1.65 | 3304 | 2.10 |
| L-LDL-C | 3627 | 1.79 | 3307 | 2.01 |
| L-LDL-CE | 3628 | 1.76 | 3306 | 2.04 |
| L-LDL-FC | 3622 | 1.92 | 3315 | 1.78 |
| L-LDL-TG | 3621 | 1.95 | 3310 | 1.93 |
| M-LDL-P | 3629 | 1.73 | 3307 | 2.01 |
| M-LDL-L | 3628 | 1.76 | 3310 | 1.93 |
| M-LDL-PL | 3634 | 1.60 | 3307 | 2.01 |
| M-LDL-C | 3628 | 1.76 | 3308 | 1.99 |
| M-LDL-CE | 3628 | 1.76 | 3304 | 2.10 |
| M-LDL-FC | 3634 | 1.60 | 3307 | 2.01 |
| M-LDL-TG | 3621 | 1.95 | 3310 | 1.93 |
| S-LDL-P | 3631 | 1.68 | 3313 | 1.84 |
| S-LDL-L | 3636 | 1.54 | 3306 | 2.04 |
| S-LDL-PL | 3635 | 1.57 | 3309 | 1.96 |
| S-LDL-C | 3631 | 1.68 | 3310 | 1.93 |
| S-LDL-CE | 3623 | 1.90 | 3308 | 1.99 |
| S-LDL-FC | 3630 | 1.71 | 3311 | 1.90 |
| S-LDL-TG | 3628 | 1.76 | 3303 | 2.13 |
| XL-HDL-P | 3646 | 1.27 | 3340 | 1.04 |
| XL-HDL-L | 3645 | 1.30 | 3343 | 0.95 |
| XL-HDL-PL | 3649 | 1.19 | 3345 | 0.89 |
| XL-HDL-C | 3640 | 1.44 | 3340 | 1.04 |
| XL-HDL-CE | 3646 | 1.27 | 3344 | 0.92 |
| XL-HDL-FC | 3633 | 1.62 | 3338 | 1.10 |
| XL-HDL-TG | 3642 | 1.38 | 3323 | 1.54 |
| L-HDL-P | 3646 | 1.27 | 3340 | 1.04 |
| L-HDL-L | 3645 | 1.30 | 3340 | 1.04 |
| L-HDL-PL | 3648 | 1.22 | 3342 | 0.98 |
| L-HDL-C | 3644 | 1.33 | 3342 | 0.98 |
| L-HDL-CE | 3647 | 1.25 | 3342 | 0.98 |
| L-HDL-FC | 3647 | 1.25 | 3339 | 1.07 |
| L-HDL-TG | 3638 | 1.49 | 3325 | 1.48 |
| M-HDL-P | 3645 | 1.30 | 3320 | 1.63 |
| M-HDL-L | 3642 | 1.38 | 3321 | 1.60 |
| M-HDL-PL | 3645 | 1.30 | 3312 | 1.87 |
| M-HDL-C | 3640 | 1.44 | 3332 | 1.27 |
| M-HDL-CE | 3638 | 1.49 | 3330 | 1.33 |
| M-HDL-FC | 3643 | 1.35 | 3329 | 1.36 |
| M-HDL-TG | 3634 | 1.60 | 3315 | 1.78 |
| S-HDL-P | 3635 | 1.57 | 3324 | 1.51 |
| S-HDL-L | 3633 | 1.62 | 3324 | 1.51 |
| S-HDL-PL | 3635 | 1.57 | 3307 | 2.01 |
| S-HDL-C | 3625 | 1.84 | 3307 | 2.01 |
| S-HDL-CE | 3618 | 2.03 | 3317 | 1.72 |
| S-HDL-FC | 3636 | 1.54 | 3310 | 1.93 |
| S-HDL-TG | 3618 | 2.03 | 3311 | 1.90 |
| VLDL_D | 3644 | 1.33 | 3326 | 1.45 |
| LDL_D | 3625 | 1.84 | 3234 | 4.18 |
| HDL_D | 3668 | 0.68 | 3354 | 0.62 |
| Serum_C | 3638 | 1.49 | 3309 | 1.96 |
| VLDL_C | 3626 | 1.81 | 3292 | 2.46 |
| Remnant_C | 3626 | 1.81 | 3314 | 1.81 |
| LDL_C | 3625 | 1.84 | 3304 | 2.10 |
| HDL_C | 3643 | 1.35 | 3340 | 1.04 |
| HDL2_C | 3650 | 1.16 | 3338 | 1.10 |
| HDL3_C | 3631 | 1.68 | 3323 | 1.54 |
| EstC | 3640 | 1.44 | 3311 | 1.90 |
| FreeC | 3640 | 1.44 | 3306 | 2.04 |
| Serum_TG | 3611 | 2.22 | 3278 | 2.87 |
| VLDL-TG | 3601 | 2.49 | 3266 | 3.23 |
| LDL-TG | 3621 | 1.95 | 3308 | 1.99 |
| HDL-TG | 3618 | 2.03 | 3319 | 1.66 |
| TotPG | 3642 | 1.38 | 3326 | 1.45 |
| PC | 3648 | 1.22 | 3311 | 1.90 |
| SM | 3641 | 1.41 | 3312 | 1.87 |
| TotCho | 3637 | 1.52 | 3320 | 1.63 |
| ApoA1 | 3642 | 1.38 | 3328 | 1.39 |
| ApoB | 3626 | 1.81 | 3312 | 1.87 |
| TotFA | 3630 | 1.71 | 3311 | 1.90 |
| DHA | 3602 | 2.46 | 3303 | 2.13 |
| LA | 3638 | 1.49 | 3323 | 1.54 |
| FAw3 | 3616 | 2.09 | 3305 | 2.07 |
| FAw6 | 3639 | 1.46 | 3314 | 1.81 |
| PUFA | 3640 | 1.44 | 3308 | 1.99 |
| MUFA | 3611 | 2.22 | 3298 | 2.28 |
| SFA | 3622 | 1.92 | 3302 | 2.16 |
| Lac | 3516 | 4.79 | 3244 | 3.88 |
| Pyr | 3578 | 3.11 | 3288 | 2.58 |
| Cit | 3641 | 1.41 | 3302 | 2.16 |
| Glol | 3624 | 1.87 | 3293 | 2.43 |
| Ala | 3640 | 1.44 | 3338 | 1.10 |
| Gln | 3642 | 1.38 | 3320 | 1.63 |
| Gly | 3629 | 1.73 | 3328 | 1.39 |
| His | 3634 | 1.60 | 3324 | 1.51 |
| Ile | 3609 | 2.27 | 3284 | 2.70 |
| Leu | 3635 | 1.57 | 3309 | 1.96 |
| Val | 3621 | 1.95 | 3319 | 1.66 |
| Phe | 3640 | 1.44 | 3328 | 1.39 |
| Tyr | 3649 | 1.19 | 3320 | 1.63 |
| Ace | 3594 | 2.68 | 3280 | 2.81 |
| bOHBut | 3519 | 4.71 | 3184 | 5.66 |
| Crea | 3654 | 1.06 | 3329 | 1.36 |
| Alb | 3472 | 5.98 | 3158 | 6.43 |
| Gp | 3630 | 1.71 | 3296 | 2.34 |

# Supplementary Table 3: Characteristics of included Born in Bradford (BiB) participants by ethnicity

| **Characteristic** | **Total**  **(**n=7,068) | **White European**  (n=3,375) | **South Asian**  (n=3,693) | **P-value** |
| --- | --- | --- | --- | --- |
| **Age (years)** | 27.3 (0.07) | 26.7 (0.1) | 27.9 (0.1) | <0.01 |
| **BMI***  Mean, kg/m^2^  Underweight or Normal  Overweight or Obese |  | | | |
|  | 26.2 (0.1) | 26.7 (0.1) | 25.7 (0.1) | <0.01 |
|  | 2512 (41.0) | 1408 (46.6) | 1104 (35.5) | <0.01 |
|  | 3617 (59.0) | 1612 (53.4) | 2005 (64.5) |  |
| **Parity (%)**  0  1  2  ≥3 |  | | | |
|  | 2942 (41.6) | 1727 (51.2) | 1215 (32.9) | <0.01 |
|  | 2010 (28.4) | 1022 (30.3) | 988 (26.8) |  |
|  | 1123 (15.9) | 406 (12.0) | 717 (19.4) |  |
|  | 993 (14.1) | 220 (6.5) | 773 (20.9) |  |
| **GDM cases (%)** | 555 (7.9) | 163 (4.8) | 392 (10.6) | <0.01 |
| **Singleton pregnancy (%)** | 6982 (98.8) | 3328 (98.6) | 3654 (98.9) | 0.24 |
| **Smoked during pregnancy (%)** | 1148 (16.2) | 1041 (30.8) | 107 (2.9) | <0.01 |

Continuous variables are presented as means and standard errors. Categorical data are presented as counts and %. Differences in categorical variables between ethnic groups were assessed via a chi squared test. Differences between continuous variables between ethnicities was assessed by a Man-Whitney test. Numbers do not match for all variables due to missing data. BMI defined using ethnic specific cut-offs of ≤23 kg/m^2^ for overweight status in South Asians and ≤25 kg/m^2^ in White Europeans. 4.33% and 12.61% of White Europeans and South Asians were missing data on smoking respectively.

| Chromosome | Start (bp) | End (bp) |
| --- | --- | --- |
| 1 | 48000000 | 52000000 |
| 2 | 86000000 | 100500000 |
|  | 134500000 | 138000000 |
|  | 183000000 | 190000000 |
| 3 | 47500000 | 50000000 |
|  | 83500000 | 87000000 |
|  | 89000000 | 97500000 |
| 5 | 44500000 | 50500000 |
|  | 98000000 | 100500000 |
|  | 129000000 | 132000000 |
|  | 135500000 | 138500000 |
|  | 25500000 | 33500000 |
| 6 | 57000000 | 64000000 |
|  | 140000000 | 142500000 |
| 7 | 55000000 | 66000000 |
| 8 | 8000000 | 12000000 |
|  | 43000000 | 50000000 |
|  | 112000000 | 115000000 |
| 10 | 37000000 | 43000000 |
| 11 | 87500000 | 90500000 |
|  | 46000000 | 57000000 |
| 12 | 33000000 | 40000000 |
|  | 109500000 | 112000000 |
| 20 | 32000000 | 34500000 |

# Supplementary Table 4: Regions of high linkage disequilibrium (LD) excluded from PCA of genetic data

#

BP: Base pair

# Supplementary Table 5: Absolute deviations in λ from 1 in each ethnicity.

| MAF cut off | White European | South Asian |
| --- | --- | --- |
| MAF <0.001 | 0.184 | 0.047 |
| 0.001≤ MAF <0.005 | 0.071 | 0.028 |
| 0.005≤ MAF <0.01 | 0.070 | 0.030 |
| 0.01≤ MAF <0.05 | 0.029 | 0.017 |
| 0.05≤ MAF <0.10 | 0.020 | 0.026 |
| 0.10≤ MAF <0.15 | 0.023 | 0.018 |
| 0.15≤ MAF <0.20 | 0.011 | 0.010 |
| 0.20≤ MAF <0.25 | 0.011 | 0.010 |
| 0.25≤ MAF <0.30 | 0.015 | 0.012 |
| 0.30≤ MAF <0.35 | 0.016 | 0.014 |
| 0.35≤ MAF <0.40 | 0.022 | 0.014 |
| 0.40≤ MAF <0.45 | 0.026 | 0.020 |
| 0.45≤ MAF <0.50 | 0.022 | 0.023 |

Average differences of λ values from 1 in each ethnicity averaged across 146 metabolite values. MAF: Minor Allele Frequency.

# Supplementary Table 6: Number of SNPs identified in each ethnicity

| Metabolite class | Metabolite | White European | | South Asian | |
| --- | --- | --- | --- | --- | --- |
|  |  | **Suggestive**  **(significant)** | **Thinned** | **Suggestive**  **(significant)** | **Thinned** |
| XXL-VLDL | XXL-VLDL-P | 25 | 4 | 1 | 1 |
|  | XXL-VLDL-L | 55 | 9 | 29 | 1 |
|  | XXL-VLDL-PL | 56 | 10 | 52 | 2 |
|  | XXL-VLDL-C | 60 | 11 | 3 | 1 |
|  | XXL-VLDL-CE | 64 | 6 | 6 | 2 |
|  | XXL-VLDL-FC | 34 | 11 | 24 | 2 |
|  | XXL-VLDL-TG | 65 | 11 | 24 | 1 |
| XL-VLDL | XL-VLDL-P | 44 | 7 | 53 | 5 |
|  | XL-VLDL-L | 42 | 10 | 41 | 2 |
|  | XL-VLDL-PL | 31 | 9 | 54 | 3 |
|  | XL-VLDL-C | 31 | 6 | 13 | 3 |
|  | XL-VLDL-CE | 26 | 7 | 17 | 4 |
|  | XL-VLDL-FC | 38 | 8 | 24 | 4 |
|  | XL-VLDL-TG | 42 | 10 | 50 | 3 |
| L-VLDL | L-VLDL-P | 11 | 3 | 58 | 4 |
|  | L-VLDL-L | 15 | 4 | 69 | 5 |
|  | L-VLDL-PL | 17 | 5 | 68 | 4 |
|  | L-VLDL-C | 21 | 7 | 79 | 3 |
|  | L-VLDL-CE | 17 | 5 | 27 | 4 |
|  | L-VLDL-FC | 17 | 6 | 39 | 4 |
|  | L-VLDL-TG | 24 | 5 | 43 | 5 |
| M-VLDL | M-VLDL-P | 26 | 5 | 53 | 1 |
|  | M-VLDL-L | 222 | 47 | 102 | 6 |
|  | M-VLDL-PL | 24 | 4 | 42 | 3 |
|  | M-VLDL-C | 35 | 9 | 26 | 1 |
|  | M-VLDL-CE | 16 | 7 | 89 | 8 |
|  | M-VLDL-FC | 226 | 35 | 85 | 3 |
|  | M-VLDL-TG | 254 | 33 | 91 | 5 |
| S-VLDL | S-VLDL-P | 52 | 5 | 24 | 3 |
|  | S-VLDL-L | 47 | 4 | 25 | 1 |
|  | S-VLDL-PL | 48 | 4 | 32 | 1 |
|  | S-VLDL-C | 35 | 5 | 2 | 0 |
|  | S-VLDL-CE | 11 | 5 | 7 | 3 |
|  | S-VLDL-FC | 49 | 4 | 33 | 2 |
|  | S-VLDL-TG | 12 | 6 | 193 | 24 |
| XS-VLDL | XS-VLDL-P | 10 | 8 | 6 | 1 |
|  | XS-VLDL-L | 16 | 8 | 9 | 1 |
|  | XS-VLDL-PL | 21 | 8 | 7 | 3 |
|  | XS-VLDL-C | 14 | 5 | 2 | 2 |
|  | XS-VLDL-CE | 23 | 9 | 1 | 1 |
|  | XS-VLDL-FC | 18 | 9 | 8 | 2 |
|  | XS-VLDL-TG | 61 | 8 | 59 | 5 |
| IDL | IDL-P | 7 | 4 | 13 | 3 |
|  | IDL-L | 6 | 3 | 11 | 2 |
|  | IDL-PL | 8 | 3 | 0 | 0 |
|  | IDL-C | 5 | 3 | 1 | 1 |
|  | IDL-CE | 10 | 6 | 7 | 1 |
|  | IDL-FC | 5 | 1 | 0 | 0 |
|  | IDL-TG | 28 | 10 | 2 | 1 |
| L-LDL | L-LDL-P | 7 | 2 | 6 | 1 |
|  | L-LDL-L | 8 | 2 | 9 | 2 |
|  | L-LDL-PL | 12 | 2 | 8 | 2 |
|  | L-LDL-C | 10 | 3 | 8 | 1 |
|  | L-LDL-CE | 13 | 3 | 8 | 1 |
|  | L-LDL-FC | 4 | 1 | 0 | 0 |
|  | L-LDL-TG | 30 | 8 | 8 | 2 |
| M-LDL | M-LDL-P | 5 | 3 | 14 | 2 |
|  | M-LDL-L | 6 | 2 | 14 | 2 |
|  | M-LDL-PL | 6 | 1 | 7 | 1 |
|  | M-LDL-C | 14 | 3 | 8 | 1 |
|  | M-LDL-CE | 14 | 3 | 0 | 0 |
|  | M-LDL-FC | 8 | 3 | 9 | 2 |
|  | M-LDL-TG | 37 | 8 | 9 | 3 |
| S-LDL | S-LDL-P | 9 | 4 | 14 | 2 |
|  | S-LDL-L | 11 | 3 | 9 | 2 |
|  | S-LDL-PL | 33 | 5 | 1 | 1 |
|  | S-LDL-C | 13 | 4 | 9 | 2 |
|  | S-LDL-CE | 19 | 4 | 0 | 0 |
|  | S-LDL-FC | 21 | 4 | 13 | 4 |
|  | S-LDL-TG | 40 | 14 | 11 | 3 |
| XL-HDL | XL-HDL-P | 76 | 10 | 8 | 2 |
|  | XL-HDL-L | 84 | 11 | 8 | 2 |
|  | XL-HDL-PL | 48 | 10 | 10 | 2 |
|  | XL-HDL-C | 52 | 13 | 5 | 1 |
|  | XL-HDL-CE | 54 | 9 | 6 | 1 |
|  | XL-HDL-FC | 41 | 9 | 12 | 3 |
|  | XL-HDL-TG | 16 | 7 | 37 | 5 |
| L-HDL | L-HDL-P | 57 | 10 | 85 | 4 |
|  | L-HDL-L | 57 | 12 | 83 | 4 |
|  | L-HDL-PL | 63 | 6 | 61 | 4 |
|  | L-HDL-C | 53 | 11 | 75 | 4 |
|  | L-HDL-CE | 52 | 11 | 8 | 4 |
|  | L-HDL-FC | 61 | 10 | 8 | 4 |
|  | L-HDL-TG | 32 | 10 | 7 | 3 |
| M HDL | M-HDL-P | 32 | 11 | 7 | 3 |
|  | M-HDL-L | 53 | 7 | 10 | 2 |
|  | M-HDL-PL | 64 | 9 | 17 | 3 |
|  | M-HDL-C | 49 | 13 | 2 | 1 |
|  | M-HDL-CE | 36 | 13 | 6 | 2 |
|  | M-HDL-FC | 74 | 12 | 13 | 3 |
|  | M-HDL-TG | 20 | 6 | 1 | 1 |
| S-HDL | S-HDL-P | 56 | 8 | 1 | 1 |
|  | S-HDL-L | 81 | 9 | 1 | 1 |
|  | S-HDL-PL | 140 | 10 | 19 | 1 |
|  | S-HDL-C | 19 | 4 | 3 | 2 |
|  | S-HDL-CE | 12 | 4 | 9 | 3 |
|  | S-HDL-FC | 76 | 9 | 3 | 2 |
|  | S-HDL-TG | 57 | 7 | 18 | 2 |
| Lipoprotein Density | VLDL_D | 39 | 7 | 20 | 3 |
|  | LDL_D | 20 | 6 | 46 | 5 |
|  | HDL_D | 74 | 12 | 31 | 4 |
| Cholesterol | Serum_C | 62 | 4 | 19 | 3 |
|  | VLDL_C | 23 | 3 | 0 | 0 |
|  | Remnant_C | 39 | 8 | 11 | 5 |
|  | LDL_C | 7 | 3 | 0 | 0 |
|  | HDL_C | 58 | 8 | 63 | 4 |
|  | HDL2_C | 67 | 9 | 31 | 6 |
|  | HDL3_C | 63 | 6 | 12 | 3 |
|  | EstC | 111 | 4 | 17 | 3 |
|  | FreeC | 23 | 3 | 9 | 2 |
| Triglycerides | Serum_TG | 19 | 4 | 34 | 3 |
|  | VLDL-TG | 17 | 7 | 50 | 3 |
|  | LDL-TG | 22 | 8 | 8 | 1 |
|  | HDL-TG | 49 | 11 | 49 | 2 |
|  | TotPG | 56 | 7 | 23 | 4 |
|  | PC | 69 | 10 | 20 | 2 |
|  | SM | 117 | 6 | 24 | 4 |
|  | TotCho | 43 | 7 | 17 | 2 |
| Apolipoproteins | ApoA1 | 58 | 10 | 32 | 6 |
|  | ApoB | 33 | 6 | 13 | 4 |
| Fatty Acids | TotFA | 40 | 3 | 8 | 3 |
|  | DHA | 33 | 5 | 4 | 1 |
|  | LA | 17 | 2 | 5 | 2 |
|  | FAw3 | 69 | 2 | 2 | 1 |
|  | FAw6 | 21 | 1 | 15 | 3 |
|  | PUFA | 26 | 1 | 20 | 4 |
|  | MUFA | 47 | 5 | 28 | 1 |
|  | SFA | 32 | 2 | 4 | 2 |
| Glycolysis Related Metabolites | Lac | 63 | 5 | 1 | 1 |
|  | Pyr | 63 | 4 | 6 | 3 |
|  | Cit | 156 | 12 | 32 | 6 |
|  | Glol | 29 | 7 | 3 | 2 |
| Unbranched Amino Acids | Ala | 156 | 14 | 53 | 2 |
|  | Gln | 5 | 2 | 2 | 2 |
|  | Gly | 15 | 8 | 9 | 2 |
|  | His | 127 | 7 | 9 | 5 |
| Branched Chain Amino Acids | Ile | 26 | 6 | 0 | 0 |
|  | Leu | 32 | 7 | 1 | 1 |
|  | Val | 6 | 4 | 8 | 3 |
| Aromatic Amino Acids | Phe | 5 | 3 | 59 | 4 |
|  | Tyr | 11 | 5 | 23 (15) | 2 |
| Ketone Bodies | Ace | 27 | 5 | 49 (1) | 4 |
|  | bOHBut | 23 | 6 | 0 | 0 |
| Fluid Balance and Inflammation | Crea | 108 | 13 | 52 | 6 |
|  | Alb | 15 | 1 | 1 | 1 |
|  | Gp | 22 | 5 | 2 | 2 |

SNPs were classified as being in LD if their R^2^ value exceeded 0.2. Suggestive level: p value≤ 1 x 10^-5^. Numbers in brackets represent the number of SNPs associated at the genome wide significant level (p value≤ 5 x 10^-8^

# Supplementary Table 7: Investigation of pleiotropy in the GWAS Catalogue and Phenoscanner databases for individual metabolite measures.

**A**

| Metabolite | SNP | GWAS Catalog | Phenoscanner | Gene |
| --- | --- | --- | --- | --- |
| Leucine | rs11586886 | - | - | - |
|  | rs11241582 | - | - | - |
|  | rs2984433 | - | BMI, Obesity class 1, weight, | ACTG1P9 |
|  | rs225598 | - | - | - |
|  | rs11270713 | - | - | - |
|  | rs73404871 | - | Mean corpuscular haemoglobin, mean corpuscular volume | TRIM56 |
|  | rs879918 | - | Self-reported Hepatitis A | - |
| HDL_D | rs13007852 | - | - | - |
|  | rs2320799 | - | - | - |
|  | rs13187167 | - | - | - |
|  | rs76030404 | - | - | - |
|  | rs153744 | - | - | - |
|  | rs55768285 | - | Height, sitting height, comparative height size at age 10, trunk fat-free mass, whole body fat free mass, arm predicted mass (left and right), whole water body mass, arm fat free mass (left) | RP11-207F8.1 |
|  | rs10899584 | - | - | - |
|  | rs996382 | - | - | - |
|  | rs55890848 | - | - | - |
|  | rs35251956 | - | Other malignant neoplasms of skin, mean corpuscular volume, self- reported malignant melanoma, red blood cell count, mean platelet volume, self-reported basal cell carcinoma | CPNE7 |
|  | rs12456654 | - | - | - |
|  | rs34733998 | - | - | - |
|  | rs16999687 | - | - | - |
| HDLC | rs17525600 | - | Stomatitis and related lesions | AC007682.1 |
|  | rs2555761 | - | - | - |
|  | rs10109848 | - | - | - |
|  | rs10219564 | - | - | - |
|  | rs7490538 | - | - | - |
|  | rs59137888 | - | - | - |
|  | rs16964949 | - | - | - |
|  | rs34733998 | - | - | - |
| HDL2C | rs77662164 | - | - | - |
|  | rs2555761 | - | - | - |
|  | rs13200375 | - | - | - |
|  | rs10109848 | - | - | - |
|  | rs10219564 | - | - | - |
|  | rs7490538 | - | - | - |
|  | rs59137888 | - | - | - |
|  | rs16964949 | - | - | - |
|  | rs34733998 | - | - | - |
| HDL3C | rs1104779 | - | - | - |
|  | rs6443637 | - | - | - |
|  | rs7490538 | - | - | - |
|  | rs7321144 | - | - | - |
|  | rs4555250 | - | - | - |
|  | rs34733998 | - | - | - |
| XS-VLDL-TG | rs11685644 | - | - | - |
|  | rs17168796 | - | - | - |
|  | rs77423873 | - | - | - |
|  | rs1573510 | - | Height | OR5AL2P |
|  | rs34447547 | Total PHF- tau (SNP x SNP interaction) | - | - |
|  | rs4781176 | - | Qualifications: college or university degree |  |
|  | rs35085155 | - | Hand grip strength left | AP000472.3 |
|  | rs28385583 | - | - | - |
| S-LDL-P | rs10915339 | - | - | - |
|  | rs77266229 | - | - | - |
|  | rs10908948 | Age at menarche | Age at menarche | UNQ6494 |
|  | rs35710612 | - | - | - |
| XL-HDL-CE | rs7766216 | - | - | - |
|  | rs2555761 | - | - | - |
|  | rs10109848 | - | - | - |
|  | rs10219564 | - | - | - |
|  | rs9510560 | - | - | - |
|  | rs7321144 | - | - | - |
|  | rs55890848 | - | - | - |
|  | rs10402413 | - | - | - |
|  | rs6126252 | - | - | - |
|  | rs35380742 | - | - | - |
| L-HDL-P | rs77662164 | - | - | - |
|  | rs2555761 | - | - | - |
|  | rs10109848 | - | - | - |
|  | rs10219564 | - | - | - |
|  | rs9510560 | - | - | - |
|  | rs7321144 | - | - | - |
|  | rs55890848 | - | - | - |
|  | rs10402413 | - | - | - |
|  | rs6126252 | - | - | - |
|  | rs35380742 | - | - | - |
| L-HDL_L | rs17525600 | - | Stomatitis and related lesions | AC007682.1 |
|  | **rs6811162** | - | Self-reported hypertension. Vascular or heart problems diagnosed by doctor: high blood pressure | ENPEP |
|  | rs77662164 | - | - | - |
|  | rs13200375 | - | - | - |
|  | rs2555761 | - | - | - |
|  | rs10109848 | - | - | - |
|  | rs10219564 | - | - | - |
|  | rs9510560 | - | - | - |
|  | rs7321144 | - | - | - |
|  | rs35163069 | - | - | - |
|  | rs6126252 | - | - | - |
|  | rs35380742 | - | - | - |
| L-HDL-C | **rs6811162** | - | Self-reported hypertension. Vascular or heart problems diagnosed by doctor: high blood pressure | ENPEP |
|  | rs6834601 | - | - | - |
|  | rs77662164 | - | - | - |
|  | rs13200375 | - | - | - |
|  | **rs55768285** | Waist circumference adjusted for BMI | Height, sitting height, comparative height size at age 10, Trunk fat-free mass, whole body fat free mass, arm predicted mass (left and right), whole water body mass, arm fat free mass (left) | LINC01621, ELOVL4 |
|  | rs2555761 | - | - | - |
|  | rs10109848 | - | - | - |
|  | rs10219564 | - | - | - |
|  | rs2028592 | - | - | - |
|  | rs35163069 | - | - | - |
|  | rs35380742 | - | - | - |
| M-HDL-C | rs6663801 | - | Comparative height size at age 10 | RP5-855F14.2 |
|  | rs10033924 | - | - | - |
|  | rs79991518 | - | - | - |
|  | rs13171149 | - | - | - |
|  | rs62443510 | - | - | - |
|  | rs62445582 | - | - | - |
|  | rs76303188 | - | - | - |
|  | rs17073913 | - | - | - |
|  | rs10085955 | - | - | - |
|  | rs904558 | - | - | - |
|  | rs12578234 | - | - | - |
|  | rs7398018 | - | - | - |
|  | rs16964930 | - | - | - |
| M-HDL-CE | rs6663801 | - | Comparative height size at age 10 | RP5-855F14.2 |
|  | rs10033924 | - | - | - |
|  | rs79991518 | - | - | - |
|  | rs62445582 | - | - | - |
|  | rs76303188 | - | - | - |
|  | rs17073913 | - | - | - |
|  | rs10085955 | - | - | - |
|  | rs17346889 | - | - | - |
|  | rs11593054 | - | - | - |
|  | rs904558 | - | - | - |
|  | rs12578234 | - | - | - |
|  | rs2138011 | - | - | - |
|  | rs7398018 | - | - | - |
| S-HDL-CE | rs11206525 | - | - | - |
|  | rs1881817 | - | - | - |
|  | rs12769447 | - | Lymphocyte count | C10orf128 |
|  | rs9795921 | - | - | - |

| Metabolite | SNP | GWAS Catalog | Phenoscanner | Gene |
| --- | --- | --- | --- | --- |
| LA | **rs12720820** | - | Self-reported high cholesterol, coronary artery disease, treatment with cholesterol lowering medication | APOB |
|  | rs721632 | - | - | - |
| FAw3 | rs7040631 | - | - |  |
| FAw6 | **rs12720820** | - | Self-reported high cholesterol, coronary artery disease, treatment with cholesterol lowering medication |  |
|  | rs11683770 | - | mDC:%32+; mDC subset (CD32+) |  |
|  | rs58865405 | - | - |  |
| M-VLDL-L | rs17028714 | - | - |  |
|  | rs62294143 | - | - |  |
|  | rs7856692 | - | - |  |
|  | rs11597600 | - | - |  |
|  | rs7224672 | - | - |  |
|  | rs73481716 | - | - |  |
| IDL-C | rs41286967 | - | - | - |
| IDL-CE | rs41286967 | - | - | - |
| L-LDL-P | rs41286967 | - | - | - |
| S-LDL-PL | rs41286968 | - | - | - |
|  | rs3814329 | - | Sitting height | RGL1 |
|  | rs10207578 | - | - | - |
| L-HDL-PL | rs6922 | - | - | - |
|  | **rs7486176** | - | Systolic blood pressure, vascular or heart problems diagnosed by doctor: high blood pressure, self-reported hypertension | C12orf76 |
| S-HDL-L | rs6490057 | - | - | - |
| S-HDL-C | rs6679531 | - | - | - |
|  | rs9938230 | - | - | - |

**B**

Bolded SNPs indicate potential confounders. **A**: Individual metabolites in white Europeans. **B**: Individual metabolites in South Asians.

# Supplementary Table 8: Associations of SNPs in Significant Instruments with Diabetes Traits in MR BASE.

**A**

| Metabolite | SNP | MR BASE diabetes disease traits |
| --- | --- | --- |
| Leucine | rs11586886 | Illness of father: diabetes, T2D with coma T1D wide definition subgroup 1 |
|  | rs11241582 | T2D, T1D, Diabetes Mellitus in pregnancy, Illness of father: diabetes |
|  | rs2984433 | T2D with neurological complications, T2D  Illness of mother: diabetes, Diabetes Mellitus in pregnancy |
|  | rs225598 | Illness of father: diabetes, Age diabetes diagnosed, T2D without complications  T2D wide definition, Unspecified diabetes, T1D wide definition, Diabetes Mellitus |
|  | rs11270713 | Started Insulin within one year of diabetes diagnosis |
|  | rs73404871 | Self-reported T2D |
|  | rs879918 | Diabetes Insipidus, T1D with ketoacidosis, T2D with coma |
| HDL_D | rs13007852 | T1D with unspecified complications, T1D with coma, Unspecified diabetes  Age diabetes diagnosed, T2D, T1D strict definition, T1D wide definition, T1D wide definition subgroup 1, E11 Non-insulin dependent diabetes mellitus, T1D with renal complications |
|  | rs2320799 | Self-reported diabetes |
|  | rs13187167 | T1D with peripheral circulatory complications, Diabetes Mellitus, E11 Non-insulin dependent diabetes mellitus |
|  | rs76030404 | T1D with renal complications, started insulin within one year of diabetes diagnosis, Insulin treated diabetes, Diabetes diagnosed by doctor, T2D, Diabetes mellitus, T2D with other conditions, T2D without complications |
|  | rs153744 | Illness of mother: diabetes |
|  | rs55768285 | - |
|  | rs10899584 | T1D with renal complications, E11 Non-insulin dependent diabetes mellitus, Diabetes Mellitus, T2D with coma |
|  | rs996382 | Illness of sibling: diabetes, T2D with ophthalmic complications, T1D |
|  | rs55890848 | Unspecific diabetes with ketoacidosis, Diabetes Mellitus in pregnancy, T2D |
|  | rs35251956 | Illness of mother: diabetes, E11 Non-insulin dependent diabetes mellitus, Diabetes Mellitus |
|  | rs12456654 | T2D without complications, T2D wide definition, illness of mother: diabetes, |
|  | rs34733998 | Diabetes diagnosed by a doctor, self-reported diabetes, T1D with ketoacidosis, T1D wide definition subgroup 1 |
|  | rs16999687 | T1D |
| HDLC | rs17525600 | Illness of father: diabetes, Diabetes diagnosed by doctor |
|  | rs2555761 | T2D |
|  | rs10109848 | T1D wide definition, subgroup 2, T2D, Unspecified diabetes |
|  | rs10219564 | Illness of siblings: diabetes, Unspecified diabetes, Eye problems related to diabetes |
|  | rs7490538 | T2D, T1D diabetes with coma, Diabetes insulin treatment, T1D ketoacidosis, T2D |
|  | rs59137888 | Diabetes insipidus, T2D, T1D, T1D with complications |
|  | rs16964949 | - |
|  | rs34733998 | Diabetes diagnosed by doctor, Self-reported diagnosis, T1D wide definition, T1D with ketoacidosis |
| HDL2C | rs77662164 | Diabetes diagnosed by doctor |
|  | rs2555761 | T2D |
|  | rs13200375 | Other diabetes wide definition, T2D, Diabetes Mellitus, Diabetes Mellitus in pregnancy, T2D with other complications |
|  | rs10109848 | T1D wide definition, subgroup 2, T2D, Unspecified diabetes |
|  | rs10219564 | Illness of siblings: diabetes, Unspecified diabetes, Eye problems related to diabetes |
|  | rs7490538 | T2D, T1D diabetes with coma, Diabetes insulin treatment, T1D ketoacidosis, T2D |
|  | rs59137888 | Diabetes insipidus, T2D, T1D, T1D with complications |
|  | rs16964949 | - |
|  | rs34733998 | Diabetes diagnosed by doctor, Self-reported diagnosis, T1D wide definition, T1D with ketoacidosis |
| HDL3C | rs1104779 | Diabetes diagnosed by doctor, T2D wide definition |
|  | rs6443637 | Diabetes related comorbidities |
|  | rs7490538 | T2D, T1D diabetes with coma, Diabetes insulin treatment, T1D ketoacidosis, T2D |
|  | rs7321144 | T1D, illness of sibling: diabetes, T2D, T2D with ophthalmic complications |
|  | rs4555250 | T2D with neurological complications, T2D with coma, diabetes diagnosed by doctor |
|  | rs34733998 | Diabetes diagnosed by doctor, Self-reported diagnosis, T1D wide definition, T1D with ketoacidosis |
| XS-VLDL-TG | rs11685644 | Diabetes diagnosed by doctor, Self-reported diabetes, Diabetes Mellitus |
|  | rs17168796 | T1D wide definition, T1D with ketoacidosis, T1D strict definition, T1D without complications, illness of sibling: diabetes, Self-reported diabetes |
|  | rs77423873 | T1D, Unspecified diabetes, T1D without complications, T2D with renal complications, Diabetes Mellitus in pregnancy, T1D with other conditions, Gestational Diabetes |
|  | rs1573510 | T1D, E11 Non-insulin dependent diabetes mellitus, Age diabetes diagnosed, Gestational Diabetes, Illness of father: diabetes, Diabetes Mellitus |
|  | rs34447547 | Illness of mother: diabetes |
|  | rs4781176 | T1D , T1D without complications, Illness of father: diabetes, T1D with neurological complications |
|  | rs35085155 | Self-reported diabetes, T2D, T2D without complications, Diabetes diagnosed by doctor, Diabetes related comorbidities |
|  | rs28385583 | T2D, T1D without complications, Unspecified diabetes with ketoacidosis, , E11 Non-insulin dependent diabetes mellitus, Diabetes Mellitus, T2D with peripheral circulatory complications |
| S-LDL-P | rs10915339 | T1D with ophthalmic complications, T1D with coma, T1D with renal complications |
|  | rs77266229 | Diabetes related comorbidities, Gestational Diabetes, Diabetes Mellitus in pregnancy |
|  | rs10908948 | Started insulin within one year of diabetes diagnosis, T1D with ketoacidosis |
|  | rs35710612 | T1D, T2D with ketoacidosis, T2D with coma, T2D with other conditions |
| XL-HDL-CE | rs77662164 | Diabetes diagnosed by doctor |
|  | rs2555761 | T2D |
|  | rs10109848 | T1D wide definition, subgroup 2, T2D, Unspecified diabetes |
|  | rs10219564 | Illness of siblings: diabetes, Unspecified diabetes, Eye problems related to diabetes |
|  | rs9510560 | T1D, T2D, T2D with other complications, T1D with coma, T1D with ketoacidosis |
|  | rs7321144 | T1D, illness of sibling: diabetes, T2D, T2D with ophthalmic complications |
|  | rs55890848 | Unspecified diabetes with ketoacidosis, Diabetes Mellitus in pregnancy |
|  | rs10402413 | Diabetes insipidus, Unspecified diabetes with ketoacidosis, T1D with ophthalmic complications, T1D |
|  | rs6126252 | T2D, Illness of father: diabetes |
|  | rs35380742 | Diabetes diagnosed by doctor, self-reported diabetes, T1D with ketoacidosis |
| L-HDL-P | rs77662164 | Diabetes diagnosed by doctor |
|  | rs2555761 | T2D |
|  | rs10109848 | T1D wide definition, subgroup 2, T2D, Unspecified diabetes |
|  | rs10219564 | Illness of siblings: diabetes, Unspecified diabetes, Eye problems related to diabetes |
|  | rs9510560 | T1D, T2D, T2D with other complications, T1D with coma, T1D with ketoacidosis |
|  | rs7321144 | T1D, illness of sibling: diabetes, T2D, T2D with ophthalmic complications |
|  | rs55890848 | Unspecified diabetes with ketoacidosis, Diabetes Mellitus in pregnancy |
|  | rs10402413 | Diabetes insipidus, Unspecified diabetes with ketoacidosis, T1D with ophthalmic complications, T1D |
|  | rs6126252 | T2D, Illness of father: diabetes |
|  | rs35380742 | Diabetes diagnosed by doctor, self-reported diabetes, T1D with ketoacidosis |
| L-HDL-L | rs17525600 | Illness of father: diabetes, Diabetes diagnosed by doctor |
|  | rs6811162 | Diabetes diagnosed by doctor, Self-reported T2D, T2D with coma, T1D |
|  | rs77662164 | Diabetes diagnosed by doctor |
|  | rs13200375 | Other diabetes wide definition, T2D, Diabetes Mellitus, Diabetes Mellitus in pregnancy, T2D with other complications |
|  | rs2555761 | T2D |
|  | rs10109848 | T1D wide definition, subgroup 2, T2D, Unspecified diabetes |
|  | rs10219564 | Illness of siblings: diabetes, Unspecified diabetes, Eye problems related to diabetes |
|  | rs9510560 | T1D, T2D, T2D with other complications, T1D with coma, T1D with ketoacidosis |
|  | rs7321144 | T1D, illness of sibling: diabetes, T2D, T2D with ophthalmic complications |
|  | rs35163069 | - |
|  | rs6126252 | T2D, Illness of father: diabetes |
|  | rs35380742 | Diabetes diagnosed by doctor, self-reported diabetes, T1D with ketoacidosis |
| L-HDL-C | rs6811162 | Diabetes diagnosed by doctor, Self-reported T2D, T2D with coma, T1D |
|  | rs6834601 | T2D with ophthalmic complications, T1D with ophthalmic complications, illness of mother: diabetes, Other diabetes: wide definition, T2D, T2D with other conditions, T1D with other conditions, Unspecified diabetes with ketoacidosis |
|  | rs77662164 | Diabetes diagnosed by doctor |
|  | rs13200375 | Other diabetes wide definition, T2D, Diabetes Mellitus, Diabetes Mellitus in pregnancy, T2D with other complications |
|  | rs55768285 | - |
|  | rs2555761 | T2D |
|  | rs10109848 | T1D wide definition, subgroup 2, T2D, Unspecified diabetes |
|  | rs10219564 | Illness of siblings: diabetes, Unspecified diabetes, Eye problems related to diabetes |
|  | rs2028592 | Illness of mother: diabetes, T2D with ophthalmic complications, Illness of father: diabetes, Unspecified diabetes with ketoacidosis |
|  | rs35163069 | - |
|  | rs35380742 | Diabetes diagnosed by doctor, Self-reported diabetes, T1D with ketoacidosis |
| M-HDL-C | rs6663801 | T2D with renal complications, T1D with ketoacidosis, T1D wide definition subgroup 2, Started insulin within one year of diabetes diagnosis |
|  | rs10033924 | T1D with neurological complications |
|  | rs79991518 | T2D with renal complications, Diabetes several complications, T1D wide definition subgroup 2, T2D with neurological complications, T1D with complications, T1D strict definition subgroup 2 |
|  | rs13171149 | T1D with coma, T2D, Self-reported T2D, Diabetes insipidus, Diabetes diagnosed by doctor |
|  | rs62443510 | Diabetes diagnosed by doctor, self-reported diabetes |
|  | rs62445582 | Started insulin within one year of diabetes diagnosis, Diabetes diagnosed by doctor, Self-reported diabetes |
|  | rs76303188 | Self-reported diabetes, T1D |
|  | rs17073913 | T1D, T1D with ophthalmic complications, T2D with peripheral circulatory complications |
|  | rs10085955 | T1D wide definition subgroup 2, Diabetes diagnosed by doctor |
|  | rs904558 | T2D, T2D with renal complications, Diabetes Mellitus, Diabetes diagnosed by doctor, Gestational Diabetes, E11 Non-insulin dependent diabetes mellitus |
|  | rs12578234 | Illness of mother: diabetes, Age diabetes diagnosed |
|  | rs7398018 | Started insulin within one year of diabetes diagnosis, Illness of mother: diabetes, T2D |
|  | rs16964930 | - |
| M-HDL-CE | rs6663801 | T2D with renal complications, T1D with ketoacidosis, T1D wide definition subgroup 2, Started insulin within one year of diabetes diagnosis |
|  | rs10033924 | T1D with neurological complications |
|  | rs79991518 | T2D with renal complications, Diabetes several complications, T1D wide definition subgroup 2, T2D with neurological complications, T1D with complications, T1D strict definition subgroup 2 |
|  | rs62445582 | Started insulin within one year of diabetes diagnosis, Diabetes diagnosed by doctor, Self-reported diabetes |
|  | rs76303188 | Self-reported diabetes, T1D |
|  | rs17073913 | T1D, T1D with ophthalmic complications, T2D with peripheral circulatory complications |
|  | rs10085955 | T1D wide definition subgroup 2, Diabetes diagnosed by doctor |
|  | rs17346889 | T2D, T2D with other complications |
|  | rs11593054 | Diabetes related comorbidities, Diabetes diagnosed by doctor, T2D |
|  | rs904558 | T2D, T2D with renal complications, Diabetes Mellitus, Diabetes diagnosed by doctor, Gestational Diabetes, E11 Non-insulin dependent diabetes mellitus |
|  | rs12578234 | Illness of mother: diabetes, Age diabetes diagnosed |
|  | rs2138011 | T1D diabetes wide definition subgroup 2, Diabetes ophthalmic comorbidities |
|  | rs7398018 | Started insulin within one year of diabetes diagnosis, Illness of mother: diabetes, T2D |
| S-HDL-CE | rs11206525 | Diabetes diagnosed by doctor, Self-reported T2D, T2D, Diabetes diagnosed by doctor, Diabetes related eye disorders, Self-reported diabetes, Illness of mother: diabetes, Illness of sibling: diabetes |
|  | rs1881817 | T1D with other complications, Diabetes diagnosed by doctor |
|  | rs12769447 | T1D with coma, T2D |
|  | rs9795921 | Illness of mother: diabetes, Diabetes Mellitus, Other diabetes: wide definition |

| Metabolite | SNP | MR BASE diabetes disease traits |
| --- | --- | --- |
| LA | rs12720820 | Age diabetes diagnosed, Diabetes related eye disorders, Illness of father: diabetes, T1D with peripheral circulatory complications, Self-reported diabetes, Diabetes diagnosed by doctor, T2D with unspecified conditions, T1D with ketoacidosis, T2D without complications |
|  | rs721632 | T1D with other complications, T2D, T1D, Insulin treated diabetes |
| FAw3 | rs7040631 | Diabetes related complications, Diabetes mellitus, E11 Non-insulin dependent diabetes mellitus, Diabetes diagnosed by doctors, T1D with ophthalmic complications, Insulin treated diabetes, T2D with other unspecified complications, Diabetes Mellitus |
| FAw6 | rs12720820 | Age diabetes diagnosed, Diabetes related eye disorders, Illness of father: diabetes, T1D with peripheral circulatory complications, Self-reported diabetes, Diabetes diagnosed by doctor, T2D with unspecified conditions, T1D with ketoacidosis, T2D without complications |
|  | rs11683770 | Diabetes diagnosed by doctor, Diabetes related comorbidities |
|  | rs58865405 | T2D with other complications, T2D, Other diabetes, T1D strict definition |
| M-VLDL-L | rs17028714 | Unspecified diabetes with ketoacidosis |
|  | rs62294143 | T1D with ketoacidosis, Diabetes related complications |
|  | rs7856692 | Diabetes mellitus, Diabetes related eye disorders, E11 Non-insulin dependent diabetes mellitus, T1D with renal complications, T1D with ketoacidosis, T1D wide definition |
|  | rs11597600 | T1D ketoacidosis, T1D |
|  | rs7224672 | T2D, Unspecified diabetes, Illness of sibling: diabetes |
|  | rs73481716 | Diabetes diagnosed by doctor, Diabetes several complications, T1D without complications, Self-reported diabetes, T1D with ophthalmic conditions, T1D with coma, Unspecified diabetes, Self-reported diabetes |
| IDL-C | rs41286967 | Self-reported T2D, T1D, T2D, T1D with ophthalmic conditions, T2D with other complications |
| IDL-CE | rs41286967 | Self-reported T2D, T1D, T2D, T1D with ophthalmic conditions, T2D with other complications |
| L-LDL-P | rs41286967 | Self-reported T2D, T1D, T2D, T1D with ophthalmic conditions, T2D with other complications |
| S-LDL-PL | rs41286968 | - |
| **B** | rs3814329 | T2D without complications, T1D wide definition subgroup 1, Diabetes, T1D with ophthalmic complications, Other diabetes wide definition, Diabetes Mellitus, Diabetes insulin treated, Diabetes related complications, T2D, T2D with unspecified complications |
|  | rs10207578 | T2D, T2D with other complications, Other diabetes wide definition, T1D, Started insulin within one year of diagnosis, Diabetes Mellitus |
| L-HDL-PL | rs6922 | T2D |
|  | rs7486176 | T1D, Self-reported diabetes, Diabetes diagnosed by doctor, T1D with ophthalmic conditions, T1D with other conditions |
| S-HDL-L | rs6490057 | T2D, Unspecified diabetes without ketoacidosis, Diabetes diagnosed by doctor |
| S-HDL-C | rs6679531 | T1D, Diabetes treated with insulin, T2D without complications, T1D with ophthalmic complications |
|  | rs9938230 | T2D with neurological complications, Diabetes with several complications, Diabetes related eye disorders, T2D |

Diabetes related disease traits (P-value ≤0.05) identified in MR BASE. LD tag SNPs R^2^ = 0.9. **A**: White European. **B**: South Asians

# Supplementary Table 9: Percentage of variation explained by PC1 in each metabolite class.

| Metabolite class | White Europeans | South Asians |
| --- | --- | --- |
| XS-VLDL | 88 | 91.5 |
| S-VLDL | 96.8 | 91.8 |
| M-VLDL | 98.9 | 95.6 |
| L-VLDL | 98.5 | 99.0 |
| XL-VLDL | 99.1 | 96.7 |
| XXL-VLDL | 96.7 | 95.1 |
| All VLDL classes | 91.9 | 86 |
| IDL | 85.8 | 92.1 |
| S-LDL | 98.0 | 97.5 |
| M-LDL | 95.5 | 95.3 |
| L-LDL | 92.5 | 91.7 |
| All LDL classes | 95.0 | 94.5 |
| S-HDL | 57.3 | 56 |
| M-HDL | 92.1 | 97.3 |
| L-HDL | 97.0 | 91.9 |
| XL-HDL | 95.2 | 97.8 |
| All HDL classes | 49.2 | 97.8 |
| Glycolysis Related Metabolites | 63.9 | 51.7 |
| Fatty Acids | 82.7 | 87.4 |
| Lipoprotein Density | 80.9 | 72.2 |
| Cholesterol | 69.7 | 59.7 |
| Glycerides and Phospholipids | 79.1 | 81.5 |
| Branched Amino Acids | 78.8 | 79.3 |
| Unbranched Amino Acids | 48.3 | 84.8 |

Percentage of variation explained by PC1 in the PCA analysis of each metabolite class following the removal of extreme outliers (3 x IQR). GWAS were performed on all classes with ≥ 2 metabolites where PC1 explained ≥70% of the variation.

# Supplementary Table 10: Correlation between PC1 and PC2 following outlier removal.

|  | PC1 | | PC2 | |
| --- | --- | --- | --- | --- |
| Metabolite Class | **White Europeans** | **South**  **Asians** | **White**  **Europeans** | **South Asians** |
| XXL-VLDL | 1 | 1 | 0.99 | 0.97 |
| XL-VLDL | 1 | 1 | 0.99 | 0.59 |
| L-VLDL | 1 | 1 | -0.39 | 0.89 |
| M-VLDL | 1 | 1 | 0.99 | 0.98 |
| S-VLDL | -0.99 | -0.99 | 0.99 | 0.97 |
| XS-VLDL | 1 | 1 | 1 | 1 |
| All VLDL | 1 | 1 | 1 | 1 |
| IDL | -0.83 | 1 | -1 | -0.63 |
| L-LDL | -0.79 | 1 | 0.99 | 0.53 |
| M-LDL | 1 | 1 | 1 | -1 |
| S-LDL | 1 | 1 | -0.37 | 0.98 |
| XL-HDL | 1 | 1 | 1 | 1 |
| L-HDL | 1 | -0.79 | 1 | 1 |
| M-HDL | 1 | 1 | 1 | 0.99 |
| S-HDL | 1 | 1 | 1 | 1 |
| All HDL | 0.94 | 0.94 | 1 | 0.41 |
| Lipoprotein Density | 1 | 1 | 1 | 1 |
| Cholesterols | 1 | -0.95 | 1 | 0.95 |
| Glycerides and Phospholipids | 1 | 0.97 | 1 | 0.84 |
| Fatty Acids | 1 | 1 | 0.99 | 1 |
| Branched Amino Acids | 1 | 1 | 1 | 1 |
| Unbranched Amino Acids | 1 | 1 | 1 | 1 |

Correlation between PC1 and PC2 following the removal of outliers (1.5 x IQR) and extreme outliers (3 x 1QR) in metabolite classes where PC1 explained ≥ 70 the variation in the metabolite class in at least one ethnicity.

# Supplementary Table 11: Investigation of pleiotropy in the GWAS Catalogue and Phenoscanner databases for identified metabolite classes.

| Ethnicity | Metabolite group | SNP | GWAS Catalog | Phenoscanner | Gene |
| --- | --- | --- | --- | --- | --- |
| White Europeans | XL-HDL | rs16999687 | - | - | - |
|  |  | rs34733998 | - | - | - |
|  |  | rs28446733 | - | - | - |
|  |  | rs9926366 | - | - | - |
|  |  | rs7321144 | - | - | - |
|  |  | rs10219564 | - | - | - |
|  |  | rs11237706 | - | - | - |
|  |  | **rs55768285** | Waist circumference adjusted for BMI | Height, sitting height, comparative height at age 10, trunk fat-free mass, trunk predicted mass, whole body fat-free mass, arm predicted mass (left and right), whole body water mass, arm fat-free mass (left), forced vital capacity | LINC01621, ELOVL4 |
|  |  | rs139019016 | - | - | - |
|  | M-LDL | rs5766358 | - | - | - |
|  |  | rs29863 | - | - | - |
|  |  | rs12419613 | - | - | - |
|  |  | rs2247545 | - | - | - |
|  |  | rs4489027 | - | - | - |
|  |  | rs7577530 | - | - | - |
|  |  | rs10915339 | - | - | - |
|  | S-LDL | rs5766358 | - | - | - |
|  |  | rs29863 | - | - |  |
|  |  | rs12419634 | Primary biliary cholangitis | - | POU2AF1 |
|  |  | rs62447465 |  | treatment with Berroca effervescent tablet | CDCA7L |
|  | All LDL classes | rs132057 | - | Tinnitus severity or nuisance | - |
|  |  | rs29863 | - | - | - |
|  |  | rs12419613 | - | - | - |
|  |  | rs79596299 | - | - | - |
|  |  | rs2247545 | - | - | - |
|  |  | rs4489027 | - | - | - |
|  |  | rs7577530 | - | - | - |
| South Asians | Fatty Acids | rs7159441 | - | - | - |
|  |  | rs55728495 | - | - | - |
|  |  | **rs12720820** | - | Self-reported high cholesterol | APOB |

# Supplementary Table 12: *Post-hoc* power analysis.

**A**

|  |  |  |  |  |  |  | Power (α = 0.05) | | | Power (α = 0.01) | |
| --- | --- | --- | --- | --- | --- | --- | --- | --- | --- | --- | --- |
| Analysis | **Group** | **True β** | **Obs** | **R^2^** | **Variance**  **of exposure** | **Variance**  **of Outcome** | **β from Unadjusted linear regression** | **β from MR** | **β from MR in**  **significant ethnicity** | **β from MR** | **β from MR in**  **significant ethnicity** |
| FAw3  Fasting | SA | 0.00662 | -0.00646 | 0.00552 | 0.0257 | 0.0130 | 0.05 | - | - | - | - |
|  | WE | 0.00327 | 0.0368 | 0.0139 | 0.0260 | 0.00819 | 0.05 | 0.98 | 0.99 | 0.93 | 0.97 |
| FAw6  2-hour | SA | -0.000584 | -0.0121 | 0.0176 | 0.0322 | 0.0623 | 0.05 | - | - | - | - |
|  | WE | 0.00341 | 0.0303 | 0.00633 | 0.0261 | 0.0566 | 0.05 | 0.07 | 0.25 | - | - |
| LA  2-hour | SA | -0.00128 | -0.00948 | 0.0113 | 0.0292 | 0.0624 | 0.05 | - | - | - | - |
|  | WE | 0.00335 | 0.0231 | 0.0125 | 0.0264 | 0.0567 | 0.05 | 0.34 | 0.47 | - | - |
| M-VLDL-L  2-hour | SA | -0.00333 | -0.00212 | 0.0363 | 1.34 | 0.0617 | 0.05 | - | - | - | - |
|  | WE | 0.00540 | 0.00623 | 0.237 | 0.457 | 0.0564 | 0.07 | 0.95 | 0.35 | 0.85 | 0.16 |
| IDL-C  2-hour | SA | -0.00365 | -0.0772 | 0.00556 | 0.0129 | 0.0623 | 0.05 | - | - | - | - |
|  | WE | 0.0223 | 0.0646 | 0.0191 | 0.0127 | 0.0564 | 0.05 | 0.11 | 0.97 | 0.03 | 0.9 |
| IDL-CE  2-hour | SA | -0.00615 | -0.0966 | 0.00571 | 0.00925 | 0.0623 | 0.05 | - | - | - | - |
|  | WE | 0.0315 | 0.0767 | 0.0311 | 0.00908 | 0.0566 | 0.05 | 0.05 | 1 | 0.01 | 0.99 |
| L-LDL-P  2-hour | SA | 5.51E+03 | 22.9 | 0.0220 | 3.27E-08 | 0.0625 | 1 | - | - | - | - |
|  | WE | 4.76E+03 | 17.3 | 0.0605 | 3.46E-08 | 0.0565 | 1 | 1 | 1 | 1 | 1 |
| S-LDL-PL  2-hour | SA | -0.0179 | -0.183 | 0.00549 | 0.00130 | 0.0624 | 0.05 | - | - | - | - |
|  | WE | 0.145 | 0.221 | 0.03193 | 0.00127 | 0.0566 | 0.06 | 0.38 | 1 | 0.18 | 1 |
| L-HDL-PL  2-hour | SA | 0.0187 | 0.0372 | 0.0227 | 0.00867 | 0.0624 | 0.05 | - | - | - | - |
|  | WE | 0.0163 | 0.0311 | 0.0408 | 0.00912 | 0.0566 | 0.05 | 0.33 | 0.88 | 0.15 | 0.71 |
| S-HDL-L  2-hour | SA | 0.00781 | 0.0105 | 0.00599 | 0.0116 | 0.0624 | 0.05 | - | - | - | - |
|  | WE | -0.00316 | -0.0415 | 0.0546 | 0.0115 | 0.0565 | 0.05 | 0.24 | 1 | 0.09 | 1 |
| S-HDL-C  2-hour | SA | -0.0126 | 0.00359 | 0.0176 | 0.00166 | 0.0623 | 0.05 | - | - | - | - |
|  | WE | 0.0370 | 0.0419 | 0.0245 | 0.00164 | 0.0566 | 0.05 | 0.05 | 0.33 | - | - |

|  |  |  |  |  |  |  | Power (α = 0.05) | | | Power (α = 0.01) | |
| --- | --- | --- | --- | --- | --- | --- | --- | --- | --- | --- | --- |
| Analysis | **Group** | **True β** | **Obs** | **R^2^** | **Variance**  **of exposure** | **Variance**  **of Outcome** | **β from Unadjusted linear regression** | **β from MR** | **β from MR in significant ethnicity** | **β from MR** | **β from MR in significant ethnicity** |
| Fatty Acids  2-hour | SA | 0.000693 | -0.000612 | 0.0175 | 6.86 | 0.01308 | 0.047 | - | - | - | - |
|  | WE | 0.00380 | 0.00650 | 0.0518 | 1.19 | 0.00814 | 0.028 | 1 | 1 | 1 | 1 |

**B**

| **C** |  |  |  |  |  |  | Power (α = 0.05) | | | Power (α = 0.01) | |
| --- | --- | --- | --- | --- | --- | --- | --- | --- | --- | --- | --- |
| Analysis | **Group** | **True β** | **Obs** | **R^2^** | **Variance**  **of exposure** | **Variance**  **of Outcome** | **β from Unadjusted linear regression** | **β from MR** | **β from MR in significant ethnicity** | **β from MR** | **β from MR in significant ethnicity** |
| Leucine  Fasting  2-hour | WE | -0.129 | -0.0220 | 0.0428 | 0.0161 | 0.00813 | 1 | - | - | - | - |
|  | SA | -0.0672 | -0.0117 | 0.00195 | 0.0170 | 0.0131 | 0.05 | 0.04 | 0.08 | - | - |
|  | WE | -0.00698 | -0.0143 | 0.0428 | 0.0161 | 0.0566 | 1 | - | - | - | - |
|  | SA | -0.0881 | -0.0334 | 0.00195 | 0.0170 | 0.0621 | 0.05 | 0.3 | 0.09 | - | - |
| HDL_D  Fasting  2-hour | WE | -0.00144 | 0.00367 | 0.0747 | 0.0415 | 0.00816 | 0.05 | - | - | - | - |
|  | SA | 0.00591 | 0.00626 | 0.0229 | 0.0395 | 0.0131 | 0.05 | 0.05 | 0.25 | - | - |
|  | WE | -0.00133 | 0.0211 | 0.0747 | 0.0415 | 0.0565 | 0.05 | - | - | - | - |
|  | SA | 0.0117 | 0.00682 | 0.0229 | 0.0395 | 0.0621 | 0.05 | 0.08 | 0.28 | - | - |
| HDLC  2-hour | WE | 0.00654 | 0.0140 | 0.0515 | 0.0155 | 0.0565 | 0.05 | - | - | - | - |
|  | SA | 0.00592 | 0.0223 | 0.0171 | 0.0148 | 0.06241 | 0.05 | 0.11 | 0.2 | - | - |
| HDL2C  2-hour | WE | 0.00636 | 0.0142 | 0.0584 | 0.0183 | 0.0566 | 0.05 | - | - | - | - |
|  | SA | 0.00617 | 0.00823 | 0.0200 | 0.0966 | 0.0623 | 0.05 | 0.08 | 0.82 | 0.02 | 0.62 |
| HDL3C  2-hour | WE | 0.0931 | 0.183 | 0.0381 | 0.000698 | 0.0566 | 0.05 | - | - | - | - |
|  | SA | 0.0598 | 0.0676 | 0.0676 | 0.000658 | 0.0622 | 0.05 | 0.92 | 0.71 | 0.79 | 0.47 |
| XS-VLDL-TG  2-hour | WE | 0.0625 | 0.0984 | 0.0501 | 0.00242 | 0.0564 | 0.05 | - | - | - | - |
|  | SA | 0.0230 | -0.0335 | 0.0296 | 0.00244 | 0.0619 | 0.05 | 0.33 | 0.4 | - | - |
| S-LDL-P  2-hour | WE | 8.31E+04 | 1.42E+02 | 0.0243 | 3.01E-09 | 0.0565 | 1 | - | - | - | - |
|  | SA | -1.74E+04 | -139.5 | 0.0114 | 3.06E-09 | 0.0624 | 1 | 0.05 | 0.05 | - | - |
| XL-HDL-CE  2-hour | WE | 0.0525 | 0.0745 | 0.0603 | 0.00506 | 0.0567 | 0.06 | - | - | - | - |
|  | SA | 0.0314 | -0.0102 | 0.00600 | 0.00477 | 0.0618 | 0.05 | 0.45 | 0.11 | - | - |
| L-HDL-P  2-hour | WE | 4.76E+03 | 17.3 | 0.0605 | 3.46E-08 | 0.0565 | 1 | - | - | - | - |
|  | SA | 5.51E+03 | 22.9 | 0.0220 | 3.27E-08 | 0.0625 | 1 | 0.31 | 0.29 | - | - |
| L-HDL-PL  2-hour | WE | 0.00732 | 0.0225 | 0.0722 | 0.0226 | 0.0565 | 0.05 | - | - | - | - |
|  | SA | 0.00857 | 0.0297 | 0.0218 | 0.0214 | 0.0625 | 0.05 | 0.31 | 0.28 | - | - |
| L-HDL-C  2-hour | WE | 0.0129 | 0.0214 | 0.0669 | 0.0138 | 0.0565 | 0.05 | - | - | - | - |
|  | SA | 0.00149 | 0.0296 | 0.0223 | 0.0130 | 0.0623 | 0.05 | 0.2 | 0.21 | - | - |
| M-HDL-C  2-hour | WE | -0.0357 | -0.0592 | 0.06553 | 0.00503 | 0.00814 | 0.07 | - | - | - | - |
|  | SA | -0.0184 | -0.0253 | 0.00614 | 0.00991 | 0.01304 | 0.05 | 0.15 | 0.12 | - | - |
| M-HDL-CE  2-hour | WE | -0.0450 | -0.0680 | 0.0362 | 0.00398 | 0.00814 | 0.06 | - | - | - | - |
|  | SA | -0.0240 | -0.0314 | 0.0114 | 0.00629 | 0.0130 | 0.05 | 0.16 | 0.3 | - | - |
| S-HDL-CE  2-hour | WE | 0.0370 | 0.0419 | 0.0245 | 0.00164 | 0.0566 | 0.05 | - | - | - | - |
|  | SA | -0.01257 | 0.00359 | 0.01757 | 0.00166 | 0.06233 | 0.05 | 0.08 | 0.6 | - | - |

**D**

|  |  |  |  |  |  |  | Power (α = 0.05) | | | Power (α = 0.01) | |
| --- | --- | --- | --- | --- | --- | --- | --- | --- | --- | --- | --- |
| Metabolite Class | **Group** | **True β** | **Obs** | **R^2^** | **Variance**  **of exposure** | **Variance**  **of Outcome** | **β from Unadjusted linear regression** | **β from MR** | **β from MR in significant ethnicity** | **β from MR** | **β from MR in significant ethnicity** |
| XL-HDL  2-hour | WE | -0.00536 | -0.00886 | 0.0585 | 0.4555 | 0.0565 | 0.06 | - | - | - | - |
|  | SA | 0.00192 | 0.00120 | 0.0109 | 2.57 | 0.0623 | 0.05 | 0.45 | 1 | 0.23 | 1 |
| S-LDL  Fasting  2-hour | WE | 0.000358 | -0.000274 | 0.0227 | 1.57 | 0.00814 | 0.05 | - | - | - | - |
|  | SA | 0.000358 | 0.000672 | 0.0171 | 0.964 | 0.0131 | 0.05 | 0.92 | 1 | 0.78 | 7 |
|  | WE | -0.00363 | -0.00694 | 0.0227 | 1.57 | 0.0565 | 0.05 | - | - | - | - |
|  | SA | -0.00215 | 0.00662 | 0.0171 | 0.964 | 0.0622 | 0.05 | 0.19 | 1 | 0.78 | 0.98 |
| M-LDL  Fasting | WE | -1.39E-04 | 0.000253 | 0.0409 | 2.69 | 0.00814 | 0.05 | - | - | - | - |
|  | SA | 0.000672 | 0.000405 | 0.00572 | 2.25 | 0.0131 | 0.05 | 0.58 | 0.58 | - | - |
| All LDL  Fasting | WE | -1.26E-04 | 0.000219 | 0.0409 | 5.19 | 0.00814 | 0.05 | - | - | - | - |
|  | SA | -0.000492 | -0.000285 | 0.00138 | 4.10 | 0.0131 | 0.05 | 0.05 | 0.06 | - | - |

Tables showing *post-hoc* MR calculation from mRND (https://shiny.cnsgenomics.com/mRnd/). Observational associations were obtained from linear regression models adjusted for maternal age (years), BMI (continuous), smoking status, multiple pregnancy, parity, and gestational age. Initial true β estimates were obtained from unadjusted linear regression models.. Additional power analyses were performed in the non-significant ethnicity to determine the power to predict the β estimate obtained from the MR analyses and the power to detect the β estimate from the significant model in the alternative ethnicity. If the power from either analysis exceeded 80% then power was also calculated for α = 0.01. Obs: β from adjusted observational studies **A:** Individual metabolite analysis in South Asians. **B**: Analysis of metabolite classes in South Asians. **C:** Analysis of Individual metabolites in White Europeans. **D:** Analysis of metabolite classes in White Europeans.
